# Topology-aware few-shot segmentation improves rice leaf-cell boundary and count fidelity, while correction is risky under tested domain shifts

**DOI:** 10.3389/fpls.2026.1950986

**Published:** 2026-10-13

**Authors:** Gaixing Wei, Da Zeng, Shenglin Qin, Dezhen Zeng

**Affiliations:** School of Big Data and Artificial Intelligence, Nanning College of Technology, Nanning, Guangxi, China

**Keywords:** domain shift, few-shot segmentation, morphometric fidelity, plant cell microscopy, rice leaf, segment anything, topology

## Abstract

**Introduction:**

Plant-cell measurements require adjacent cells to remain separated, yet few-shot segmentation is usually assessed mainly by foreground overlap.

**Methods:**

We developed domain-aware topology prompt adaptation (DTPA), which trains a small topology head over a frozen microscopy-specific Segment Anything backbone. We evaluated matched 10-shot segmentation on a leakage-free RiceCell split, conducted a secondary eight-image audit, and assessed label-free routing and selective correction on locked external tests.

**Results:**

DTPA improved boundary F1 and reduced cell-count error relative to ConvNeXt, while reducing Dice. The boundary advantage persisted across one- to five-pixel tolerances and against two matched instance baselines. The secondary audit linked this trade-off to improved cell-count and typical-cell-area fidelity, but not to every image-derived measurement. The label-free router reduced severe under-counting on a locked PlantSeg root test. Selective correction was less reliable under the tested tissue shifts: 14 of 45 accepted Ovules retries were harmful, and the only accepted floral-meristem retry was harmful.

**Discussion:**

Topology-aware prompting and routing are computational tools for plant microscopy, not evidence of biological discovery or universal model superiority. A correction-abstention rule derived after the external audit is only a deployment proposal and requires prospective target-domain validation.

## 1 Introduction

Cell-resolved plant microscopy supports quantitative studies of tissue organisation, growth and morphology. Such measurements require accurate cell counts, continuous walls, and faithful estimates of area, shape, and neighbourhood structure. A single merge or split can distort all of these quantities, yet instance annotations that preserve this detail are expensive. Widely used biomedical architectures, including U-Net and nnU-Net, perform well when representative labels are available (Ronneberger et al., 2015; Isensee et al., 2021). Instance-oriented systems such as Cellpose, StarDist, and Omnipose can also separate crowded or irregular cells (Schmidt et al., 2018; Stringer et al., 2021; Cutler et al., 2022). Plant tissues present additional challenges, including tightly packed cells, large-scale differences, and acquisition-dependent wall contrast. PlantSeg addresses these challenges through learned boundary maps and graph partitioning in three-dimensional tissues (Wolny et al., 2020). Retraining a task-specific network remains difficult when only a few independent images are labelled.

Promptable foundation models provide an alternative. Segment Anything (SAM) (Kirillov et al., 2023), MedSAM (Ma et al., 2024), and the microscopy-specific *μ*SAM (Archit et al., 2025) offer reusable encoders and flexible point or mask prompts. CellSAM extends this approach to automatic cell segmentation (Marks et al., 2025). CelloType jointly addresses object detection, segmentation, and classification (Pang et al., 2025). Simulation-based inference instead adds an explicit morphological prior (Jones et al., 2025). Cellpose-SAM is a recent general-purpose cell model (Pachitariu et al., 2025).

Automatic prompting introduces distinct failure modes. Prompt density may be poorly matched to cell scale, several prompts may retrieve the same object, or one positive prompt may span adjacent cells. Prompt construction must therefore be treated as part of the segmentation problem. Background-centric few-shot prompting constrains over-segmentation (Bo et al., 2026), structural prompts align semantic focus with target parts (Xia et al., 2026), and domain-adaptive SAM variants modify features or adapters (Wang and Xiao, 2024; Zhu et al., 2026). Efficient unsupervised prompts and preference optimisation reduce annotation dependence (Konwer et al., 2025). UNICL-SAM weights in-context examples by uncertainty (Sheng et al., 2025). SAMIX retrieves semantic reference masks under mixed supervision (Hu et al., 2026), whereas Point Prompt Defender suppresses harmful point prompts during inference (Liu et al., 2026). An unresolved challenge is to combine few-shot plant-cell topology, label-free prompt-scale routing, and explicit assessment of selective-correction risk.

Evaluation presents a separate problem. Dice and intersection over union (IoU) measure foreground agreement but can hide changes in connectivity and cell count. Boundary losses (Karimi and Salcudean, 2020; Kervadec et al., 2021), persistent-homology objectives (Hu et al., 2019; Clough et al., 2022), and clDice (Shit et al., 2021) address this mismatch directly. The CVPR 2026 SCNP method similarly penalises misclassified neighbouring pixels to improve topology (Valverde et al., 2026). For compact plant cells, important errors include broken boundaries, duplicate masks, false merges, false splits, and incorrect counts.

We asked whether a frozen microscopy SAM backbone could recover cell boundaries and counts from a few *independent* rice leaf images. The study addressed three questions. First, can a small topology adaptation head improve boundary and count accuracy under a matched annotation budget? Second, can label-free routing avoid transferring an unsuitable pixel scale to a new tissue? Third, can a selective-correction controller reject harmful negative-prompt retries? **Figures 1** and **2** map these questions to the topology adaptation head, domain-shift router, and two-gate controller. **Table 1** links each component to its testable evidence and evidential limits. Our main contribution is a topology-conditioned prompt field in which adaptation, routing, and correction can be audited separately. We evaluated domain-aware topology prompt adaptation (DTPA) through matched-budget comparisons, an eight-image human review, component ablations, and locked cross-tissue stress tests. The intended plant-science contribution is a computational measurement tool: reliable wall placement and instance separation are prerequisites for cell counts, projected area, and neighbourhood summaries in tissue images. We therefore test whether segmentation preserves those measurements, rather than claiming a rice developmental or physiological discovery. This design distinguishes matched-domain gains from correction risks in the tested domain shifts.

**Figure 1:**
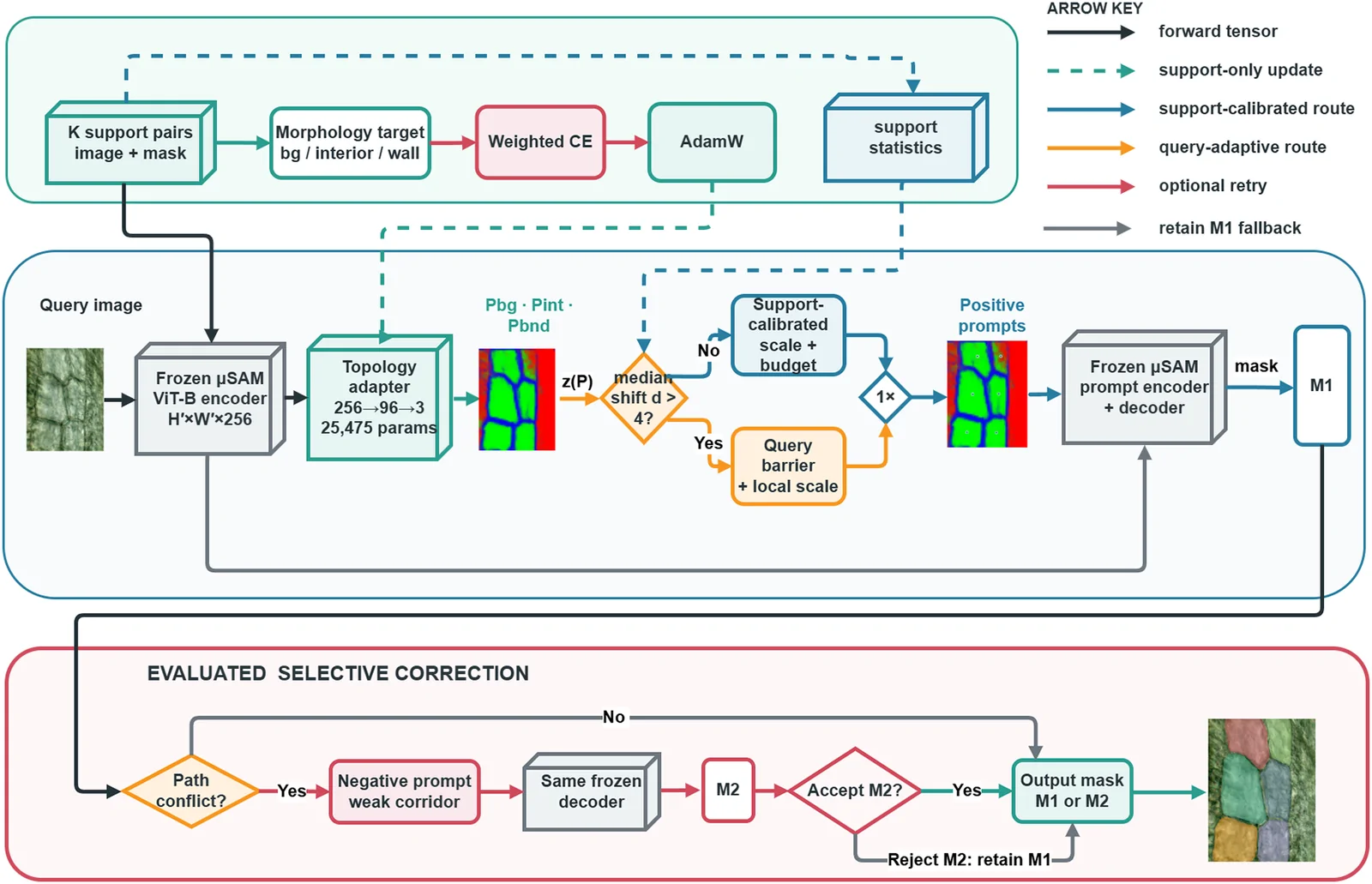
Overall DTPA architecture. The detailed support-calibration path retains the morphology target, weighted cross-entropy loss, AdamW update, and frozen support statistics; training updates only the 25,475-parameter topology adaptation head. A label-free domain-shift score selects exactly one prompt route before frozen decoding. The evaluated correction path retains the path-conflict decision, negative-prompt retry, candidate $M_{2}$, acceptance decision, and explicit $M_{1}$ fallback. Solid charcoal denotes forward tensor flow; dashed teal denotes support-only calibration; orange denotes mutually exclusive route selection; and blue denotes prompts and candidate masks. The RiceCell trace links the query image, topology field, selected prompts, and final instances. The untested post-audit domain-level abstention proposal has been removed from this architecture figure so that no unevaluated safeguard is shown as part of the tested pipeline.

**Figure 2:**
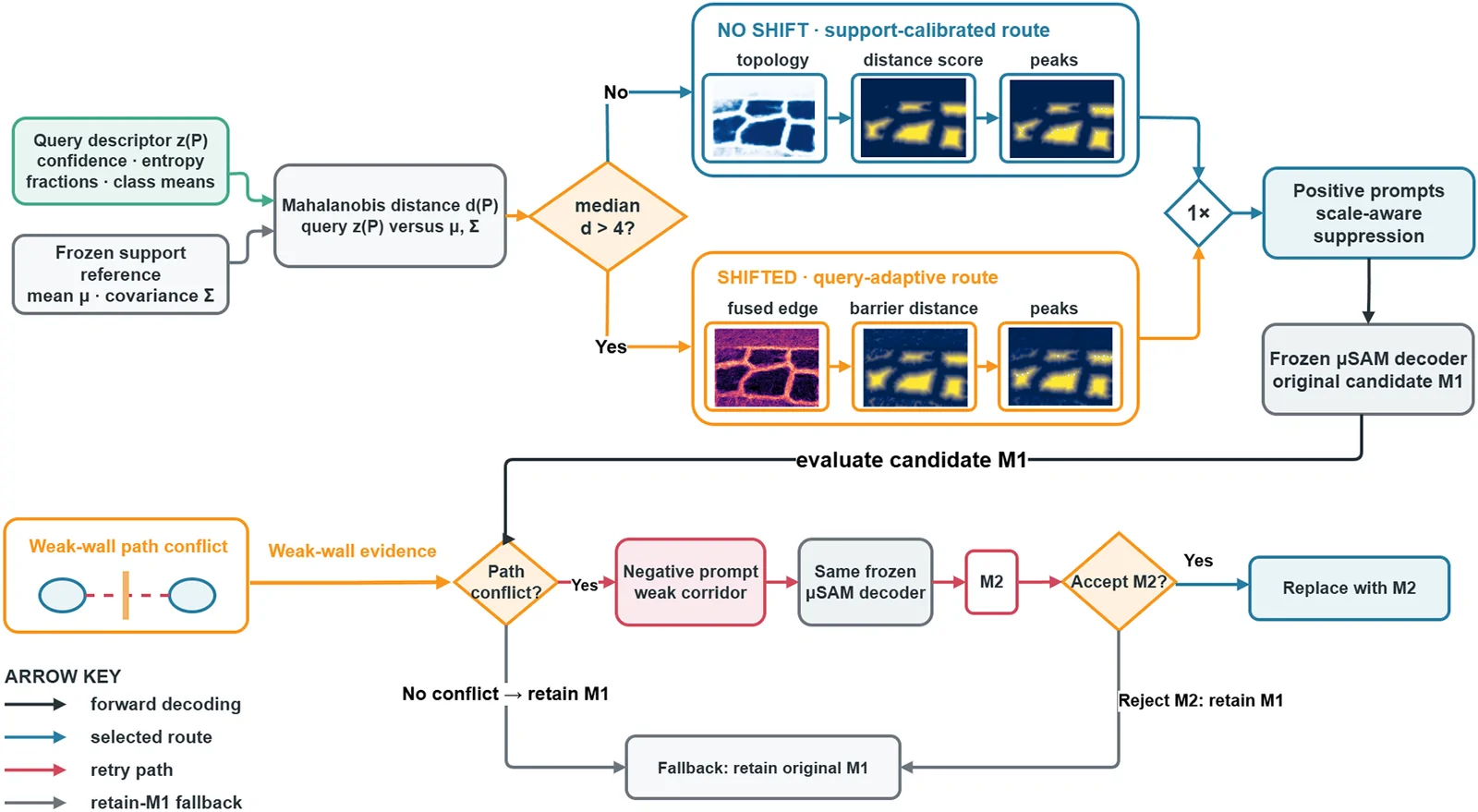
Exclusive prompt routing and evaluated selective correction. The upper flow calculates the label-free Mahalanobis shift score, selects exactly one prompt route, and decodes the original candidate $M_{1}$. The lower flow evaluates a weak-wall path conflict, generates at most one negative-prompt retry $M_{2}$, and replaces $M_{1}$ only if the acceptance gate passes; every failed branch returns $M_{1}$. Black denotes forward decoding, blue the selected route, red the retry path, and grey the fallback. Only the evaluated controller is shown; the post-audit domain-level abstention proposal has been removed from the schematic and remains described solely as unvalidated future work.

**Table 1:** Mechanism-to-evidence map.

Problem	Design element	Operational effect	Evidence and boundary
Few independent labels	25,475-parameter topology adaptation head on frozen μSAM features	Adapts the interior/boundary prompt field while preserving the backbone	Matched support selections improve boundary F1 and cell-count MAE but reduce Dice
Domain shift	Domain-shift router selects either the support-calibrated or query-adaptive route	Avoids applying one pixel scale to every domain	PlantSeg root improves, while route selection and split/merge behaviour remain domain dependent
Ambiguous prompt retry	Path-conflict gate, acceptance gate, and original-mask fallback	Separates retry activation from mask replacement	Two validation events are improvements, but 14 Ovules harms and one floral-meristem harm preclude a general safety claim
Binary-mask proxy bias	Family-level split and candidate-blinded two-reader audit	Separates reference uncertainty from topology performance	Agreement is 83.3%; the proxy misses 5/73 instances, and the reference remains candidate-assisted

Each component is linked to a failure assumption, a testable result, and the corresponding evidential limit. External failures are retained.

### 1.1 Related work

#### 1.1.1 Cell and plant-tissue segmentation

Marker-controlled watershed remains widely used to separate touching objects (Vincent and Soille, 1991). Modern cell-segmentation systems instead learn markers or spatial flows from image features. StarDist predicts distances to star-convex object boundaries (Schmidt et al., 2018). Cellpose predicts spatial flows and can be specialised with limited user data (Stringer et al., 2021; Pachitariu and Stringer, 2022). Omnipose relaxes the strong shape constraint for bacterial morphology (Cutler et al., 2022). PlantSeg combines a convolutional boundary predictor with graph partitioning for cellular-resolution plant volumes (Wolny et al., 2020). DTPA is not intended to replace these fully supervised systems. It adapts automatic prompts when only a few independent rice image families are available.

#### 1.1.2 Few-shot and promptable segmentation

Prototype-based few-shot segmentation transfers class representations from a support set to a query image (Wang et al., 2019). Generalised few-shot formulations must also retain performance on previously observed classes (Tian et al., 2022). SAM changes the segmentation interface by accepting spatial prompts (Kirillov et al., 2023). *μ*SAM adapts this interface to microscopy and supports automatic instance segmentation (Archit et al., 2025). CellSAM provides a foundation model for automatic cell segmentation (Marks et al., 2025). Low-rank adaptation (LoRA) enables parameter-efficient feature tuning (Hu et al., 2022). SAMDA addresses domain adaptation for SAM (Wang and Xiao, 2024). DTPA instead preserves the frozen microscopy SAM backbone and adapts the *prompt field*. Topology probabilities determine prompt locations, candidate-mask plausibility, and whether the support-derived pixel scale should be used.

#### 1.1.3 Topology and boundary reliability

Boundary-aware objectives address class imbalance and surface error (Karimi and Salcudean, 2020; Kervadec et al., 2021). Persistent-homology losses explicitly match connected components and holes (Hu et al., 2019; Clough et al., 2022). clDice emphasises skeleton agreement in tubular structures (Shit et al., 2021). SCNP introduces a computationally efficient local-neighbour penalty that improves topological accuracy across segmentation frameworks (Valverde et al., 2026). Plant-cell segmentation differs from tracing a single tubular network because numerous compact components must remain distinct. We use interior and boundary evidence to control automatic prompts and report count, split, and merge errors alongside region overlap.

## 2 Materials and methods

### 2.1 Problem setup

Let the support set be $S={\left\{(x_{i},y_{i})\right\}}_{i=1}^{K}$, where K∈{1,2,5,10,20} counts independent acquisition or augmentation families rather than transformed views of one image. Each $y_{i}$ is a binary rice-cell mask. From this mask, we derive a three-class topology target $t_{i}(p)\in \{0,1,2\}$ for background, interior, and a radius-3 morphological boundary. The goal is to predict an instance map for a query image x without updating the light-microscopy μSAM ViT-B backbone.

#### 2.1.1 Intended use and study scope

DTPA is designed for measurement-oriented segmentation from a few independently sourced plant-cell masks. It targets wall continuity, instance separation, cell count, and image-derived morphology. Treatment classification and biological mechanism are outside its scope. Accordingly, this study examines segmentation accuracy, measurement preservation, and transfer failure, not a developmental or physiological hypothesis in rice.

### 2.2 DTPA separates learning, routing, and decoding

DTPA changes the prompt field while leaving the microscopy SAM backbone and mask decoder frozen (**Figure 1**). The support set has two roles: it trains the topology adaptation head and defines the reference distribution for domain-shift detection. For each query, the frozen encoder produces a dense feature map that the adaptation head converts into background, interior, and boundary probabilities. A label-free router selects one of two prompt-generation routes, after which the frozen decoder converts the selected positive prompts into candidate instances. The controller replaces the original candidate mask $M_{1}$ only when both the path-conflict and acceptance gates pass. This evaluated pipeline contains no domain-level correction gate. The design keeps topology learning, cross-domain prompt adjustment, and mask selection as separate operations.

We evaluated the routed segmentation pipeline and the selective-correction controller separately. The pipeline produces $M_{1}$ through topology adaptation, exclusive route selection, prompt generation, and duplicate suppression. The controller can then generate $M_{2}$, but replacement still requires both gates to pass. A routing improvement therefore provides no evidence that correction is beneficial, while a failed correction does not negate an upstream routing gain. We describe a domain-level abstention rule separately as a future safeguard because it was formulated only after the external audit.

**Figure 3** shows the only trainable network module. **Figure 2** then combines routing, prompt generation, and selective correction into one decision sequence.

**Figure 3:**
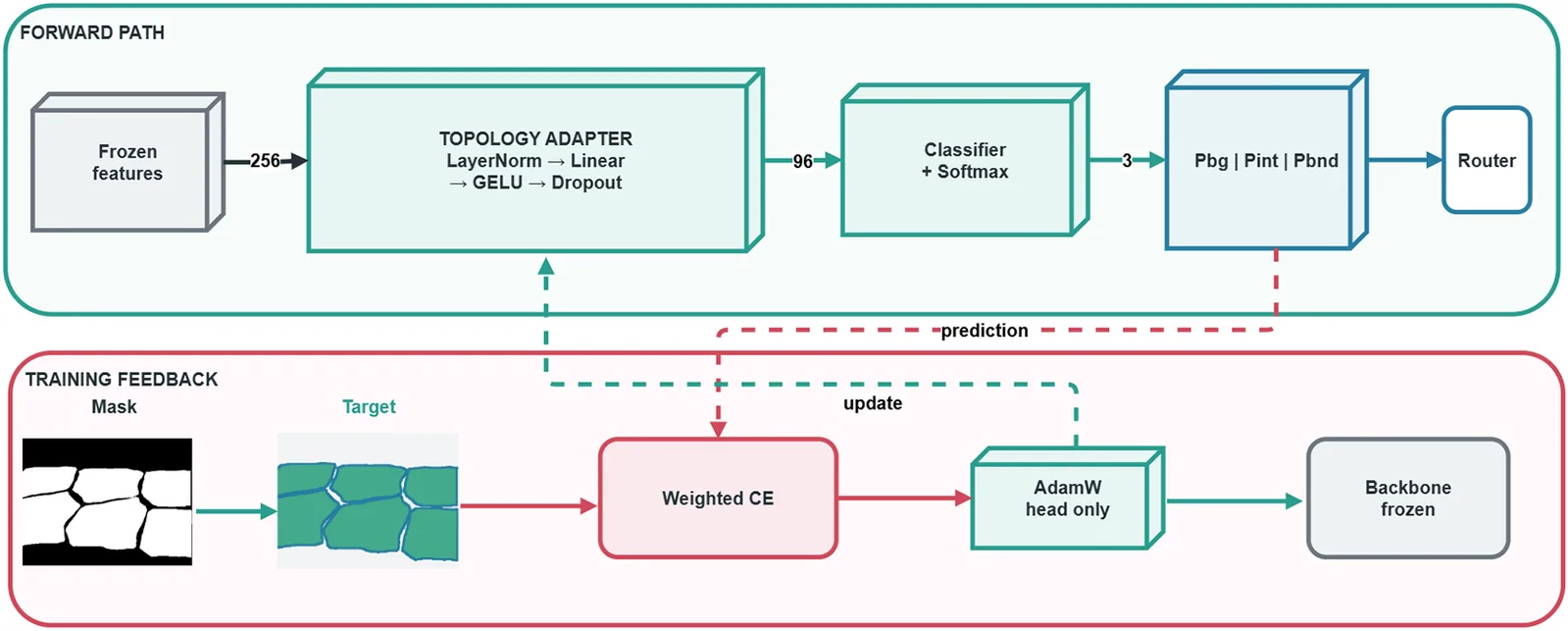
Topology adaptation head. Each spatial feature vector passes through layer normalisation, two pointwise linear projections (equivalent to 1×1 convolutions), GELU, and dropout. The head uses no spatial pooling, so the $H^{\prime }\times W^{\prime }$ grid is preserved before bilinear upsampling. A class-weighted loss supervises background, interior, and three-pixel boundary classes. AdamW updates only the topology adaptation head; the encoder and decoder remain frozen. The embedded channels show how binary support masks provide spatial boundary supervision.

### 2.3 The topology adaptation head separates cell interiors from boundaries

The topology adaptation head converts frozen $F(x)\in {\mathbb{R}}^{H^{\prime }\times W^{\prime }\times 256}$ features into three probability fields (**Figure 3**; Equation 1):

(1) $$F\to LN_{256}\to Linear_{256\to 96}\to GELU\to Dropout_{0.1}\to Linear_{96\to 3}\to {↑}_{bilinear}\to P$$

The linear layers act independently at each spatial location and are equivalent to 1×1 convolutions. Pooling is not used, so the feature-grid resolution is unchanged. The topology adaptation head was trained for 300 epochs with the loss defined in Equation 2:

(2) $$L_{topo}=-\sum_{p}\;\sum_{c=0}^{2}w_{c}\text{𝟙}[t(p)=c]logP_{\theta }(c|F(x{)}_{p})$$

where the inverse-frequency class weights are clipped to [0.5,5]. AdamW used a learning rate of $2\times 10^{-3}$, weight decay of $10^{-3}$, cosine annealing, and no more than 8,192 sampled feature-grid locations per epoch. At each epoch, locations were drawn uniformly without replacement from the pooled support pixels; if fewer than 8,192 were available, all were used. The sample was redrawn every epoch by a run-specific pseudorandom generator. This algorithm and sample cap were identical across support selections; only the support identities and registered run seed changed.

### 2.4 The domain-shift router prevents indiscriminate scale transfer

The development chronology contained two distinct stages. First, a prespecified single-pass potato experiment exposed failure of the fixed support scale and motivated adding a domain-shift router. Potato labels, outcomes, and descriptor values were not subsequently used to fit the reference distribution, select descriptor terms, choose candidate routes, or set the routing threshold. The router itself does not use target labels. Each topology map P was summarised by an eight-dimensional descriptor z(P): mean maximum probability, mean normalised entropy, three hard-class fractions, and three mean class probabilities. We estimated the support mean μ and covariance Σ from these descriptors, adding a $10^{-5}I$ ridge term. The domain-shift score was defined by Equation 3:

(3) $$d(P)=\sqrt{{\left(z(P)-\mu \right)}^{⊤}\Sigma^{†}(z(P)-\mu )}$$

Routing was applied once per dataset. A median image score above the frozen threshold of 4.0 selected the query-adaptive route. This threshold was chosen from RiceCell support and validation data and PlantSeg development data before the PlantSeg root test labels were downloaded. Thus, potato motivated the concept but did not tune the threshold or the reported route decisions.

### 2.5 Prompt synthesis adapts cell scale by domain

The support-calibrated route converts interior probabilities into conservative positive prompts. Candidate pixels require high interior probability and low boundary probability. Their distance transform was weighted by $P_{int}-0.35P_{bnd}$. Median equivalent radius, area quantiles, and cell count in the support set determined peak spacing, the valid mask-area range, and the prompt budget (Equation 4):

(4) $$B_{s}=\text{min}(40,\lceil 1.5\text{max}({\tilde{N}}_{s},A_{f}/{\tilde{A}}_{s})+1\rceil )$$

RiceCell used the support-calibrated route.

The query-adaptive route estimates cell scale from the query image. A robustly scaled Scharr gradient of the contrast-normalised greyscale image provided $E_{img}$, while a smoothed $P_{bnd}-0.35P_{int}$ map provided the topology edge. We formed $E=0.42E_{img}+0.58E_{topo}$, thresholded the top 24% of responses and morphologically closed them to form barriers. We then computed a distance transform over the traversable field. For a traversable pixel p, the query candidate field is defined by Equation 5:

(5) $$U_{q}(p)=D_{E}(p)[0.45+0.55T(p)][1-0.35E_{\text{img}}(p)]$$

The topology term is defined by Equation 6:

(6) $$T(p)={\left[P_{\text{int}}(p)-0.45P_{\text{bnd}}(p)\right]}_{+}$$

where $D_{E}$ is the barrier-constrained distance transform. Local maxima of $U_{q}$ defined candidate prompts, while their distance radii provided a query-specific estimate of typical cell area. Radius-dependent non-maximum suppression retained at most 80 prompts.

The prompt coefficients were development heuristics rather than fitted model parameters. We selected the 0.35 topology contrast, the 0.42/0.58 image–topology mixture, and the top-24% barrier quantile while inspecting RiceCell support/validation and PlantSeg development behaviour, then froze them before the external tests. We retained the 80-prompt ceiling because the factorial development experiment showed severe under-coverage at 24 and 40 prompts on dense PlantSeg slices. A post-review one-factor-at-a-time analysis varied the image weight from 0.25 to 0.60, the barrier quantile from 0.70 to 0.82, and the topology contrast from 0.25 to 0.45 with correction disabled. This sensitivity analysis evaluates local dependence on the heuristics; it is not a second round of external-test tuning.

### 2.6 The selective-correction controller limits mask replacement

SAM returned three masks for each positive prompt. We removed masks outside the area range defined by the selected route and scored each remaining candidate using Equation 7:

(7) $$s_{\text{cand}}=s_{\text{SAM}}+0.60{\bar{P}}_{\text{int}}-0.80f_{\text{bnd}}-0.18\text{min}(4,n_{\text{enc}})$$

where $f_{\text{bnd}}$ is the fraction of mask pixels with $P_{\text{bnd}}>0.46$, and $n_{\text{enc}}$ counts other prompt centres enclosed by the mask. Candidates were ranked by $s_{\text{cand}}$. Any mask with an IoU above 0.58 relative to a retained candidate was suppressed.

Overlap alone cannot identify a prompt error because two prompt centres may belong to different cells separated by a true wall. We therefore scored the line corridor $C_{ij}$ between candidate centre $q_{i}$ and each competing centre $q_{j}$. After excluding the endpoints, we defined corridor evidence from the fused image -topology edge field E using Equation 8:

(8) $$w_{ij}=1/2Q_{0.90}(E{|}_{C_{ij}})+1/2max(E{|}_{C_{ij}}),\kappa_{ij}=1-w_{ij}$$

A high $\kappa_{ij}$ indicates weak boundary evidence between two competing prompts. The path-conflict gate triggered a negative-prompt retry only when the mean score ${\bar{\kappa }}_{i}$ reached the validation-frozen threshold of 0.60. The decoder then received at most four competing centres as negative prompts.

Removing a competing point did not by itself justify mask replacement. We computed the acceptance score using Equation 9:

(9) $$s_{\text{acc}}=0.32r_{\text{resolved}}+0.22{\bar{\kappa }}_{i}+0.18r_{\text{SAM}}+0.16r_{\text{topo}}+0.12r_{\text{area}}$$

where the five normalised terms quantify conflict removal, path support, SAM score retention, interior–boundary consistency, and the plausibility of the $M_{2}$-to- $M_{1}$ area ratio. The acceptance gate replaced $M_{1}$ only when the retry removed a conflict, the area ratio lay within [0.30,1.08], and $s_{\text{acc}}\geq 0.45$. Both thresholds were selected on PlantSeg validation data and then frozen. We used target labels only in a retrospective audit that classified accepted events as improvements, harms, or neutral outcomes; the labels never entered the inference gate. For this audit, let I(m) denote the best IoU between mask m and any reference instance, and let d(m) count the reference instances for which m covers at least 10% of the instance area. We defined the general event oracle using Equation 10:

(10) $${\text{Δ}}_{\text{evt}}(\lambda )=[I(m_{\text{new}})-I(m_{\text{old}})]+\lambda [d(m_{\text{old}})-d(m_{\text{new}})]$$

The main audit used λ=0.10. An accepted event was an improvement when ${\text{Δ}}_{\text{evt}}>\epsilon$, a harm when ${\text{Δ}}_{\text{evt}}<-\epsilon$, and neutral otherwise. The main analysis used ϵ=0.01. Neither parameter affected inference.

The non-negative score weights sum to one and encode the development-time priority order: actual conflict removal, wall-path evidence, retention of the decoder score, topology consistency, and area plausibility. The path, acceptance, and area thresholds were selected on PlantSeg validation data and then frozen. A post-review retrospective analysis on the locked Ovules event record removed one score term at a time and varied both the acceptance threshold and admissible area range. These analyses diagnose dependence of the observed failures on the hand-specified rules; they do not retune the deployed controller.

#### 2.6.1 Proposed future deployment safeguard (not evaluated)

This post-audit proposal lies outside the validated method. Routing and correction require different responses to domain shift. A dataset median ${\tilde{d}}_{D}$ above 4.0 selects query-adaptive prompts but does not authorise mask replacement. The locked external audit led us to formulate the domain-level correction gate as follows (Equation 11):

(11) $${ɡ}_{corr}(D)=\text{𝟙}[{\tilde{d}}_{D}\leq 4.0]\text{𝟙}[U_{0.95}(R_{h})\leq 0.10]\text{𝟙}[C\geq 0.10]$$

where $U_{0.95}(R_{h})$ is the upper one-sided 95% Wilson bound for harm risk, and C is the acceptance coverage on a held-out target-domain calibration set. When calibration data are unavailable, either risk criterion fails, or ${\tilde{d}}_{D}>4.0$, $g_{\text{corr}}=0$. The routed pipeline continues, but all retries abstain and return $M_{1}$. The 10% risk and coverage budgets are conservative post-audit specifications. They did not alter any reported experiment, and the tested pipeline did not apply this gate. Ovules and floral-meristem results were obtained with the original frozen controller. Those results motivate the proposal but provide no prospective validation of it.

### 2.7 Experimental design and evaluation

#### 2.7.1 Datasets and leakage control

##### 2.7.1.1 RiceCell

The supplied collection contains 680 image–mask pairs. We first grouped numeric identifiers by decade and then calculated exact SHA-256 hashes for every image and thresholded mask under the eight dihedral transforms: rotations of 0, 90, 180, and 270 degrees, horizontal and vertical reflections, transpose, and anti-transpose. Families were the union of these exact image or mask matches, including cross-index matches. Near matches were not merged automatically, and no subjective adjudication was used. Within each family, a deterministic lexicographic score based on black-pixel fraction, intermediate-label fraction, portrait orientation, brightness distance from 125, negative sharpness, and numeric identifier selected the base image. The procedure reduced 680 pairs to 82 families. A final cross-split hash check found no exact dihedral match after the fixed-seed allocation of 58/12/12 families to training/validation/test.

The main analysis used one base image per family; offline augmentations were not counted as independent shots. Binary masks were thresholded at 127. For count-based metrics, connected components used 8-connectivity and a minimum area of 30 pixels. These connected components are proxy instances: a small gap can split one cell and an absent wall can merge neighbours. They averaged only 7.67 components per test image and therefore limit every count- or instance-based claim from the binary reference. DTPA, ConvNeXt and LoRA+decoder used the same nested support identities. Five support selections were tested at 1, 2, 5, and 20 shots, and ten at 10 shots.

##### 2.7.1.2 Potato

The public potato tuber microscopy dataset contributed 60 bright-field image–mask pairs (Biswas and Barma, 2020). The archive, pair list, preprocessing procedure, and support-scale topology prompt adaptation checkpoint (TPA-v1) were locked before evaluation. This single-pass cross-crop failure test revealed that support-scale prompting did not transfer and motivated the router concept. Potato labels, outcomes, and descriptor values were not used to select router features, tune prompt parameters, or choose the threshold of 4.0.

##### 2.7.1.3 PlantSeg root

PlantSeg provides official root training, validation, and test volumes (Wolny et al., 2020). Development used 126 standardised two-dimensional training slices and 24 validation slices. Before obtaining the test archive, we registered the raw-image slice rule, intensity normalisation, and aspect-preserving resize to a 512-pixel long side. The checkpoint hash, domain-shift threshold, prompt ceilings, and metrics were also fixed. The locked test set contains 48 slices from four volumes. Because its labels tessellate the tissue, foreground Dice is weakly informative; instance metrics were therefore emphasised. Root endpoints remain secondary in the study-wide hierarchy. Test labels, overlays and per-image scores were not inspected before the single-pass evaluator was frozen.

##### 2.7.1.4 PlantSeg ovules

We preregistered the official seven-volume Ovules archive as a second locked cross-tissue test before downloading or reading its arrays. We included every x– y slice with a positive raw-image 99th-to-1st percentile range, thereby applying a label-blind selection rule. Configuration D, the routed pipeline through duplicate suppression, was compared with configuration G, which adds the path-conflict and acceptance gates (**Supplementary Table S6**). Both used the frozen path threshold of 0.60 and acceptance threshold of 0.45. The three-dimensional volume, not the slice, was the independent statistical unit. Because Ovules belongs to PlantSeg, it tests cross-tissue shift but not independent-laboratory acquisition.

##### 2.7.1.5 Independent floral meristems

We used University of Cambridge time-series images of *Arabidopsis* floral meristems with expert instance labels (Refahi et al., 2021; Kar et al., 2022). Before downloading the pixel data, we fixed the archive checksum, checkpoint and threshold hashes, preprocessing, metrics, and a label-blind slice rule. The rule used only stack dimensions to select the median axial slice from each of ten published time points. These data differ from RiceCell in species, organ, laboratory, and acquisition series. The time points are nevertheless nested within only two biological series, FM1 and FM6. We treated the dataset as a locked stress test, not as evidence of broad domain generalisation.

##### 2.7.1.6 Units of analysis

After duplicate merging, independent acquisition or augmentation families formed the RiceCell support and test units. We summarised PlantSeg root and Ovules results by volume, while floral-meristem cases remained nested within two biological series. Images, slices, and segmented cells were not treated as biological replicates. The study compares computational methods and transfer behaviour rather than group-level plant phenotypes.

#### 2.7.2 Endpoint hierarchy

The reporting hierarchy prevents small, correlated metric families from receiving equal evidential weight (**Table 2**). The two co-primary endpoints were 10-shot RiceCell boundary F1 and cell-count mean absolute error (MAE) under matched support, with Dice serving as a secondary guardrail for region overlap. We did not interpret gains in the co-primary topology endpoints as overall superiority when Dice decreased. This hierarchy was formalised during manuscript revision rather than registered prospectively. It limits claim strength but does not convert support-selection tests into confirmatory population inference.

**Table 2:** Endpoint hierarchy and permitted claims.

Tier	Endpoints	Analysis unit	Permitted interpretation
Primary	RiceCell 10-shot boundary F1 and cell-count MAE	Ten matched support selections on locked test families	Topology-oriented few-shot performance in the matched RiceCell setting
Secondary	RiceCell Dice/IoU; audit-subset segmentation accuracy (SEG), instance F1 at IoU 0.5, boundary F1, count and median area; locked PlantSeg root SEG and cell-count MAE	Matched support selection, or image for descriptive audit; locked test slice/volume	Region-topology trade-off, measurement fidelity and routed transfer
Exploratory	Other shot counts, shape and adjacency descriptors, corruption, ablations, efficiency, Ovules/floral event oracles, and calibration analyses	Support selection, audit image, validation slice or external volume/series	Mechanism diagnosis, sensitivity, and failure boundary; no general efficacy claim

Primary endpoints define the matched-domain claim. Secondary endpoints assess trade-offs and measurement effects, while exploratory endpoints examine mechanisms and transfer limits.

#### 2.7.3 Baselines and training protocol

The equal-budget trainable instance baseline combined rank-4 LoRA with a trainable automatic instance-segmentation decoder (LoRA+decoder AIS) (Hu et al., 2022; Archit et al., 2025). It was trained for 250 iterations with $384^{2}$ patches and a learning rate of $10^{-4}$. Five preregistered support selections were used at 1, 2, 5, and 20 shots, and ten at 10 shots. The ten 10-shot sets covered 52 of 58 training families. Their pairwise overlap ranged from zero to five families (median, two), and no family appeared in more than four sets. Each run used the same support and test families as DTPA. Connected components from the same binary masks provided the instance targets, so the baseline required no additional manual annotation. A separate repeat review quantified bias in these proxy instances. We use the name “LoRA+decoder” because its optimiser state contained 9.74 M trainable parameters rather than only the low-rank matrices.

Frozen μSAM AIS/APG, StarDist, and Cellpose-SAM served as zero-shot contextual references rather than equal-budget baselines. Cellpose 4.2.1.1 cpsam_v2 was run once with frozen defaults, automatic diameter estimation, and no target-domain tuning (Pachitariu et al., 2025). StarDist 0.9.2 used the official 2D_versatile_fluo weights (Schmidt et al., 2018). Its scale and probability threshold were selected on RiceCell validation data using instance $F1_{0.5}$ and then fixed at 0.25 and 0.30 for the locked test. CellSAM was integrated from the official release, but its weights required an unavailable access token. It is reported as not evaluated rather than given a surrogate score (Marks et al., 2025).

For a target-trained specialist comparison, we initialised StarDist from the official 2D_versatile_fluo weights and fine-tuned it separately on each of the same ten 10-shot support selections used by DTPA and LoRA+decoder. Full-resolution images were converted to greyscale and normalised to the 1st–99.8th percentiles. Each run used eight epochs, eight steps per epoch, batch size two, the inherited learning rate of $3\times 10^{-4}$, and random rotations and reflections. The checkpoint with the lowest validation loss was retained. The 12 fixed validation families then selected probability and non-maximum-suppression thresholds separately for each run before one evaluation on the 12 locked test families. This post-review experiment matches the support identities, repeat count, and annotation budget, but each support selection still has only one optimiser seed.

The conventional baseline used an ImageNet-pretrained ConvNeXt-Tiny encoder (Liu et al., 2022) and a trainable boundary feature pyramid network (Boundary-FPN) decoder. Training combined Dice, binary cross-entropy, and boundary objectives with AdamW, cosine decay, and validation-based early stopping. Of 29.63 M total parameters, 1.81 M were trainable. Independent runs were assigned across two RTX 4090 graphics processing units (GPUs).

LoRA+decoder AIS is the equal-budget trainable instance baseline; Cellpose-SAM and the original StarDist run supply frozen zero-shot context. The additional 10-shot StarDist result is an exploratory target-trained specialist reference. Target-trained CellSAM and PlantSeg models were not tested, so the study does not claim superiority over all trainable instance-segmentation systems.

We also separated support-selection variation from training stochasticity in a post-review replication. All five runs for each trainable method used the same repeat-0 10-shot support families and the original training configuration; only the registered run seed, and hence parameter initialisation or stochastic training order and augmentation, changed. We report the mean, sample standard deviation, and range across these five fixed-support optimiser-seed runs. This analysis complements, rather than replaces, the ten matched support selections.

The prompt-construction factor study used all 24 PlantSeg validation slices. We disabled negative-prompt retry but retained duplicate suppression to isolate upstream design choices from selective correction. The query-adaptive route followed a 2×3×3 design: support- or query-derived scale; image, learned-topology, or fused barriers; and prompt ceilings of 24, 40, or 80. Three support-calibrated controls used the same prompt ceilings. We report all 21 configurations, route contrasts at matched budgets, and query-route marginal means. Image-level bootstrap intervals describe sensitivity without treating correlated configurations as biological replicates.

#### 2.7.4 Metrics and statistics

RiceCell metrics were foreground Dice, IoU, boundary F1 at a three-pixel tolerance, and absolute cell-count error from connected-component proxy instances. Boundary F1 was calculated on the native image grid from the foreground union of each final instance map. A (3) morphological gradient defined the predicted and reference boundaries, and symmetric dilation supplied the stated pixel tolerance. We calculated one score per locked test family and then used an equal-weight mean across the 12 families for each support selection. The **Figure 4** ConvNeXt boundary series was reconstructed from the immutable checkpoints under this rule because its original training log summarised padded evaluation batches. The DTPA and LoRA+decoder source metrics already used native-image, per-family evaluation. We additionally recalculated boundary F1 at tolerances of one to five pixels on every matched 10-shot prediction. PlantSeg metrics were segmentation accuracy (SEG), instance F1 at an IoU threshold of 0.5, boundary F1, cell-count MAE, and split and merge events per image. An undirected cell-adjacency graph was constructed after Hungarian matching at a minimum IoU of 0.10 and a two-pixel wall-gap tolerance. Graph metrics included adjacency precision, recall and F1, degree MAE, and normalised edge error.

**Figure 4:**
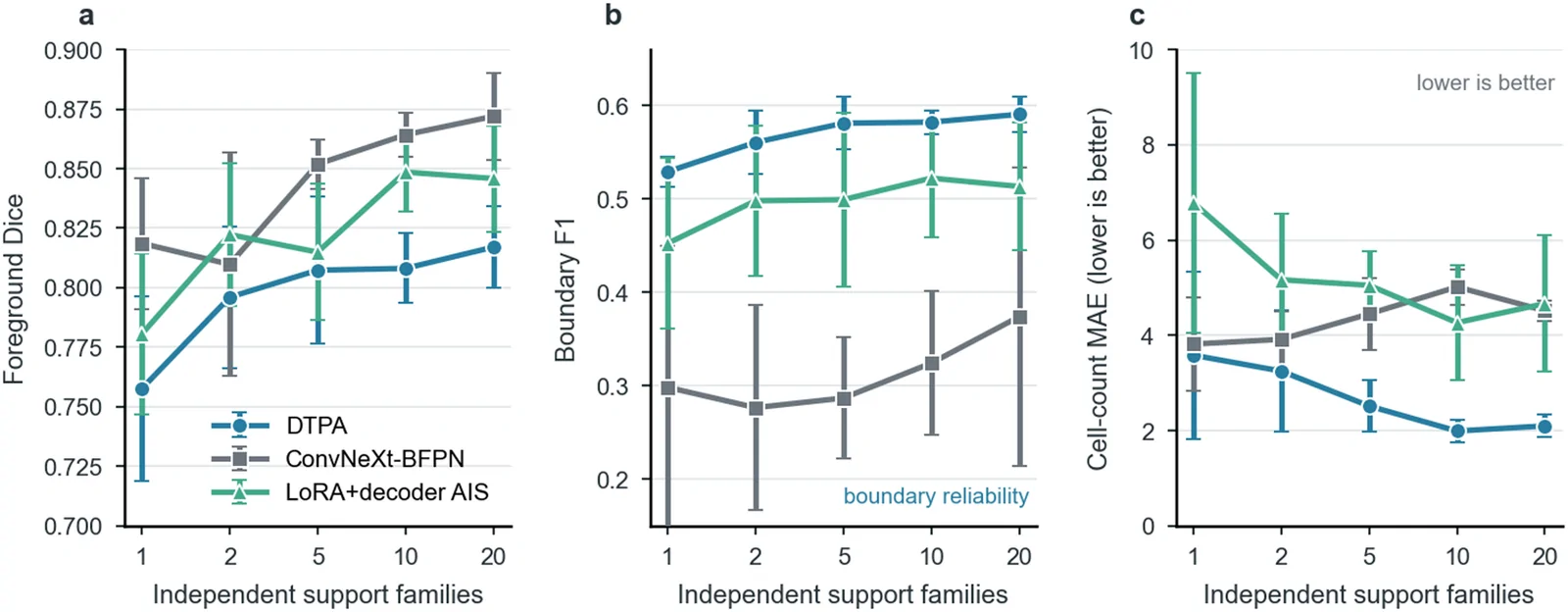
Leakage-free RiceCell few-shot results. Points and error bars show the mean ± sample standard deviation across matched support selections (DTPA, ConvNeXt–Boundary-FPN, and LoRA+decoder AIS: n=10 at 10 shots and n=5 otherwise). They do not include independent optimiser-seed variability. Boundary F1 uses the native-grid, per-family result stream defined in Methods; the ConvNeXt series was reconstructed from immutable checkpoints under that single evaluation rule. **(a)** Foreground Dice; **(b)** boundary F1; **(c)** cell-count MAE (lower values are better). The error bars describe support-selection variability and do not imply population-level inference. Values and reconstruction provenance are supplied with the frozen source tables.

Selective-correction events were recorded as triggered, accepted, improvement, harm, or neutral. The all-trigger ledger contains one observation-level row for each of the 93 Ovules triggers reconstructed from the locked shard JSON files. A valid triggered retry required the decoder to return a finite, non-empty replacement candidate for the triggered positive prompt. Event metrics_shard_1:N_590_final_crop_ds2:z0151:p073:e00 returned no such mask (rejection reason: no-valid-retry). We excluded this event before acceptance scoring and oracle classification, leaving 92 valid triggered retries. Improvement and harm were retrospective oracle outcomes that combined the change in best target-instance IoU with a penalty for spanning multiple target cells. These outcomes were never used for inference.

We conducted two distinct analyses with the eight retained human-reviewed RiceCell images. The first compared the prespecified 20-shot, repeat-0 DTPA prediction with zero-shot StarDist. The eight images were descriptive units, and image-level bootstrap intervals summarised sensitivity within this audit set. The matched 10-shot analysis evaluated DTPA, ConvNeXt, and LoRA+decoder against the same eight references for each of ten matched support selections. We first averaged each measurement error across the fixed eight images within each method and support selection. We then treated the ten matched support-selection summaries as the bootstrap units. Neither analysis contributed to the co-primary endpoints. For each instance map, we calculated cell count, total projected foreground area, median cell area in pixels, and median shape factor (Equation 12):

(12) $$\phi =\frac{4\pi A}{P_{C}^{2}}$$

where A is instance area and $P_{C}$ is the Crofton perimeter. Labels were expanded by three pixels across narrow wall gaps to form an undirected neighbourhood graph, from which mean degree was calculated. For image j, total projected-area and median cell-area errors were defined by Equation 13:

(13) $$E_{\text{proj},j}=\frac{\left|A_{j}^{\text{pred}}-A_{j}^{\text{ref}}\right|}{A_{j}^{\text{ref}}},E_{\text{med},j}=\frac{\left|{\tilde{A}}_{j}^{\text{pred}}-{\tilde{A}}_{j}^{\text{ref}}\right|}{{\tilde{A}}_{j}^{\text{ref}}}$$

Shape-factor and mean-degree errors were absolute differences. We report all eight image-level values for the descriptive audit and all ten matched support-selection summaries for the matched analysis. These images were audit cases, not biological replicates, and lacked physical calibration. The analysis assesses measurement preservation rather than a rice phenotype.

Score selectivity was audited across the N valid triggered retries after ranking the composite scores $s_{i}$. For a retrospective threshold τ, the accepted set and coverage are defined by Equation 14:

(14) $$A_{\tau }=\{i:s_{i}\geq \tau \},\;C\left(\tau \right)=\frac{\left|A_{\tau }\right|}{N}$$

The harm risk is defined by Equation 15:

(15) $$R_{h}(\tau )=\frac{\sum_{i\in A_{\tau }}\text{𝟙}[y_{i}=h]}{\left|A_{\tau }\right|}$$

The benefit precision is defined by Equation 16:

(16) $$P_{+}(\tau )=\frac{\sum_{i\in A_{\tau }}\text{𝟙}[y_{i}=+]}{\left|A_{\tau }\right|}$$

Here, C denotes coverage, $R_{h}$ harm risk, and $P_{+}$ benefit precision. The score-only curve is retrospective because the deployed controller also enforces conflict-reduction and area-ratio constraints. Scores were divided into five equal-frequency bins $B_{b}$ to calculate the empirical benefit frequency in each bin (Equation 17):

(17) $${\hat{p}}_{b}^{+}=|B_{b}{|}^{-1}\sum_{i\in B_{b}}\text{𝟙}[y_{i}=+]$$

The diagnostic expected calibration error (ECE) is defined by Equation 18:

(18) $$\text{EC}{\text{E}}_{\text{diag}}=\sum_{b}\frac{\left|B_{b}\right|}{N}|{\bar{s}}_{b}-{\hat{p}}_{b}^{+}|$$

This error is diagnostic because $s_{acc}$ is a composite score, not a learned probability.

For repeated RiceCell runs, tables report the mean and sample standard deviation across matched support selections ( n=5, except n=10 at 10 shots). Paired mean differences, 10,000-resample bootstrap intervals, and exact two-sided Wilcoxon tests described sensitivity to support selection. These analyses were exploratory. At other shot counts, n=5 limited the smallest non-zero two-sided Wilcoxon value to 0.0625. Holm adjustment was applied to Dice, boundary F1, and cell-count MAE within each shot count. We do not treat these tests as confirmatory population inference. Their error bars capture support-selection variability only. The separate five-run fixed-support analysis reports optimiser-seed sensitivity descriptively and does not change the analysis unit of the matched support-selection experiment. The frozen seed registry lists every numeric training seed, bootstrap seed, resampling count, bit generator, stream-initialisation rule, and rule for undefined bootstrap samples.

Controller comparisons used paired image-level bootstrap intervals, Wilcoxon signed-rank tests, and Holm adjustment within each metric family. Ovules comparisons were paired by volume, and confidence intervals resampled the seven volume-level differences. Slice-level values remained descriptive. For accepted correction events, we report an event-level Wilson interval for harm rate and a 50,000-resample cluster bootstrap in which the seven Ovules volumes, with all their within-volume events, were sampled with replacement. The same cluster bootstrap supplied the benefit-versus-harm area under the receiver operating characteristic curve (AUROC) interval. Floral-meristem intervals resampled the ten locked cases only to describe case variability. Because the cases were nested within two biological series, they do not support confirmatory population inference.

#### 2.7.5 Reproducibility records and execution order

We organised the revision materials as six linked record layers so that every major result can be traced from its input definition to its final output. The first records input provenance, SHA-256 identifiers, duplicate-family membership, and locked train–validation–test assignments. The second records registered support selections, model identifiers, training configurations, and the original software-environment snapshots. The third records training and bootstrap seeds, resampling counts, random-number generators, stream-initialisation rules, and the treatment of undefined resamples. The fourth links native-grid prediction records to metric definitions, analysis units, and paired or cluster-aware resampling rules. The fifth is the observation-level correction ledger, including the one triggered event with no finite non-empty retry. The sixth contains figure-source tables, expected key results, and file-level integrity manifests. The accompanying reproducibility map names the file implementing each layer and links every major result family to its script, input table, analysis unit, and expected output.

The registered execution order separates two reproducibility levels. Analytical replay starts from the distributed de-identified or aggregate source tables. It regenerates or cross-checks the matched RiceCell, routing, prompt-sensitivity, controller-risk, StarDist-summary, and optimiser-seed analyses before a package-wide integrity check. Full model reruns additionally require the original microscopy arrays and model weights. They use the archived experiment scripts, registered support identities, environment snapshots, and checkpoint or input hashes. Raw RiceCell arrays are permission-controlled and are therefore identified by hash rather than redistributed, whereas public external inputs are obtained from the cited sources. This distinction permits independent cross-checking of the reported revision analyses without implying unrestricted access to the third-party RiceCell collection.

## 3 Results

Results follow the endpoint hierarchy defined above. We first examine matched-domain boundary and count performance, then measurement fidelity and routing, and finally exploratory correction and robustness analyses.

### 3.1 Few-shot RiceCell trade-off

The methods favoured different aspects of the task (**Figure 4**). At 10 shots, DTPA achieved a higher boundary F1 than ConvNeXt ( 0.5822±0.0127 versus 0.3242±0.0771) and a lower cell-count MAE ( 1.99±0.23 versus 5.02±0.37). These were the co-primary endpoints. The paired DTPA-minus-ConvNeXt boundary-F1 difference was 0.2580 (95% bootstrap interval 0.2094–0.3060; exact two-sided Wilcoxon P=0.001953; within-shot Holm-adjusted P=0.005859). We interpret this test as support-selection sensitivity rather than population-level inference. Conversely, the secondary Dice guardrail favoured ConvNeXt ( 0.8081±0.0146 versus 0.8642±0.0091). All ten matched support selections showed the same directions. The remaining shot-specific values and paired sensitivity analyses are reported in **Figure 4** and the **Supplementary Information**. The repeats capture variation due to support selection on fixed test families; they do not measure optimiser-seed variation or establish population-level superiority.

**Table 3** summarizes RiceCell results for the equal-budget trainable instance baseline and contextual references.

**Table 3:** RiceCell results for the baselines and contextual references.

Method	Shots	Dice	Boundary F1	Cell-count MAE ↓
μSAM AIS	0	.8297	.2891	8.42
μSAM APG	0	.8294	.5013	17.67
Cellpose cpsam_v2	0	.0023	.0050	7.67
StarDist 2D_versatile_fluo	0	.7178	.3350	5.75
StarDist target-trained	10	.6623 ± .0921	.3794 ± .0386	2.90 ± .47
LoRA/DTPA	1	.7806/.7576	.4524/.5292	6.78/3.58
LoRA/DTPA	2	.8223/.7960	.4980/.5605	5.17/3.25
LoRA/DTPA	5	.8149/.8074	.4992/.5811	5.05/2.52
LoRA/DTPA	10	.8485/.8081	.5223/.5822	4.27/1.99
LoRA/DTPA	20	.8458/.8170	.5134/.5906	4.67/2.10

LoRA+decoder AIS is the equal-budget trainable instance baseline. Matched entries are means for LoRA+decoder AIS and DTPA over identical support selections (n = 10 at 10 shots and n = 5 otherwise). StarDist, Cellpose-SAM, and frozen μSAM are single-run zero-shot contextual references. The target-trained StarDist row uses the same ten 10-shot support selections. **Figure 4** reports the sample standard deviations.

RiceCell results for the baselines and contextual references. LoRA+decoder AIS is the equal-budget trainable instance baseline. Matched entries are means for LoRA+decoder AIS and DTPA over identical support selections ( n=10 at 10 shots and n=5 otherwise). StarDist, Cellpose-SAM, and frozen μSAM are single-run zero-shot contextual references. The target-trained StarDist row uses the same ten 10-shot support selections. **Figure 4** reports the sample standard deviations.

The same region–topology trade-off was present against LoRA+decoder AIS at every shot count. All 30 paired comparisons used the same support images, number of repeats, and annotation budget. The zero-shot rows are included only as contextual references.

Across the ten matched support selections, target-trained StarDist reached Dice 0.6623±0.0921, boundary F1 0.3794±0.0386, and cell-count MAE 2.90±0.47. The corresponding DTPA values were 0.8081±0.0146, 0.5822±0.0127 and 1.99±0.23. StarDist also reached SEG 0.4157±0.0812 and instance $F1_{0.5}$ 0.4356±0.0612. This repeated, equal-annotation-budget comparison strengthens the positioning against one public specialist model, but it does not establish superiority over all target-trained instance-segmentation systems or quantify independent optimiser-seed variation. Per-repeat results are reported in **Supplementary Table S10**.

The fixed-support optimiser-seed replication separated training stochasticity from support selection (**Supplementary Figure S9**; **Supplementary Table S9**). Across five seeds, DTPA reached Dice 0.8324±0.0048, boundary F1 0.5981±0.0053, and count MAE 2.30±0.07. ConvNeXt reached 0.8665±0.0029, 0.2957±0.0320, and 4.97±0.17, while LoRA+decoder reached 0.8439±0.0125, 0.4825±0.0753 and 4.13±0.69. The DTPA boundary and count directions persisted in every seed, as did the Dice cost relative to ConvNeXt. These values describe one fixed support set and the same 12 test families; they are not additional independent test samples.

### 3.2 Matched measurement consequences of the Dice trade-off

The boundary advantage did not depend on the prespecified three-pixel tolerance (**Figure 5a**). Across tolerances of one to five pixels, mean boundary F1 ranged from 0.367 to 0.696 for DTPA, from 0.188 to 0.414 for ConvNeXt, and from 0.339 to 0.608 for LoRA+decoder. Every paired bootstrap interval for DTPA minus either comparator remained above zero.

**Figure 5:**
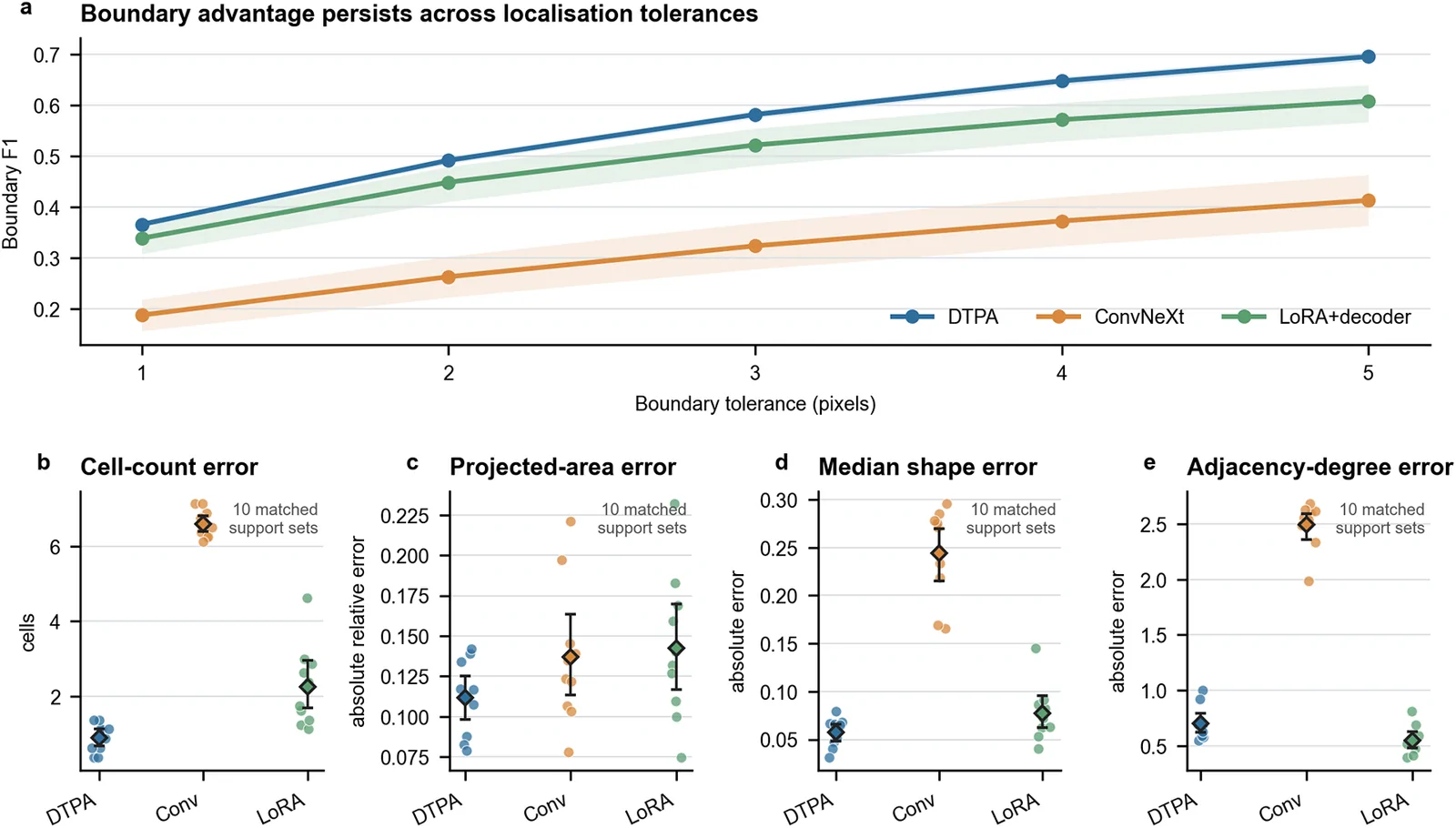
Boundary-tolerance and matched measurement sensitivity. **(a)** Boundary F1 at one- to five-pixel tolerances on all 12 locked test families. Lines are means and bands are sample standard deviations across ten matched 10-shot support selections. **(b–e)**, Per-support-selection mean errors across the eight retained human-reviewed images for cell count, total projected foreground area, median shape factor, and mean adjacency degree. Diamonds and bars show means and 95% bootstrap intervals across the ten matched support selections. Lower values are better in **(b–e)**. The human-reviewed subset is secondary and candidate-assisted.

The same ten matched 10-shot predictions were also evaluated against the eight retained human-reviewed references (**Figures 5b–e**; **Supplementary Table S4**). Mean absolute count error was 0.90 for DTPA, 6.60 for ConvNeXt, and 2.26 for LoRA+decoder. Median cell-area relative error was 0.205, 4.793, and 0.301, respectively, while median shape-factor error was 0.057, 0.244, and 0.077. Total projected-area error was similar (0.112, 0.137, and 0.143), and both paired intervals for the DTPA difference included zero. DTPA reduced adjacency-degree error relative to ConvNeXt (0.699 versus 2.492), but LoRA+decoder was lower at 0.551. The Dice reduction therefore did not erase the count, boundary, or typical-cell-area gains in this small audit, but neither did those gains extend to every downstream measurement. These eight images remain a secondary, candidate-assisted audit rather than biological replicates.

### 3.3 Secondary human-reviewed instance audit and StarDist

RiceCell contains binary masks, so connected-component labelling can join cells across small wall gaps. We ranked the 12 locked test images by this ambiguity and presented connected-component and wall-gap candidates for review (**Supplementary Figure S6**). The first reader completed two rounds before adjudicating discordant decisions. A second reader independently assessed a randomised, identity-blinded Candidate A/B package.

**Supplementary Table S2** summarises within-reader repeatability and between-reader agreement. Before review, we selected the 20-shot, repeat-0 DTPA prediction for a maximum-support secondary analysis of reference bias. This audit remained separate from the 10-shot co-primary analysis, and its results were not pooled with the primary comparison.

Adjudication retained eight images and excluded four uncertain cases. The human-reviewed reference contained 73 instances, compared with 68 in the connected-component proxy. The proxy therefore missed 5 of 73 instances and undercounted by 0.625 cells per image. Against the human-reviewed reference, DTPA exceeded StarDist in SEG (0.6402 versus 0.5251), $F1_{0.5}$ (0.7443 versus 0.4035), and boundary F1 (0.6354 versus 0.3610). This secondary audit estimates proxy-label bias. It neither replaces the locked binary-mask endpoints nor constitutes independently acquired biological ground truth.

The result was unchanged in the reference-sensitivity analysis (**Supplementary Table S7**). Across all 16 proxy/prelabel combinations for the four retained images with reader disagreement, DTPA had higher SEG, $F1_{0.5}$, and boundary F1 than StarDist, and lower absolute cell-count error. The same directions appeared in a coherent second-reader scenario that also retained G027 ( n=9). The four-image exact-consensus subset is reported as a stress test, not as an alternative reference.

### 3.4 Secondary DTPA–StarDist audit of reference bias

We next examined whether instance errors altered image-derived measurements (**Supplementary Figure S7**). Compared with StarDist, DTPA yielded lower errors in cell count (0.625 versus 6.50) and median area (0.289 versus 0.390). Adjacency-degree error was also lower (0.603 versus 0.963), whereas shape-factor error was not. The count and area directions were stable across every admissible n=8 reference and the second-reader n=9 scenario. The adjacency result remained stable in those analyses but reversed in the four-image exact-consensus subset, while shape factor was also reference sensitive. The audit therefore provides the strongest support for preserving cell count and typical area. Adjacency remains exploratory, and these image-derived measurements do not establish a biological phenotype.

### 3.5 Boundary-error overlays localise the quantitative gain

The retained images illustrate the behaviour captured by the locked-test metrics (**Figure 6**). Compared with the zero-shot contextual references and matched-support ConvNeXt and LoRA+decoder AIS baselines, DTPA recovered more of the human-reviewed boundary within the two-pixel agreement band and produced fewer isolated background contours. These examples illustrate the quantitative findings but do not form an additional test set. **Supplementary Figures S4**, **S5** show complete fields for all eight retained images.

**Figure 6:**
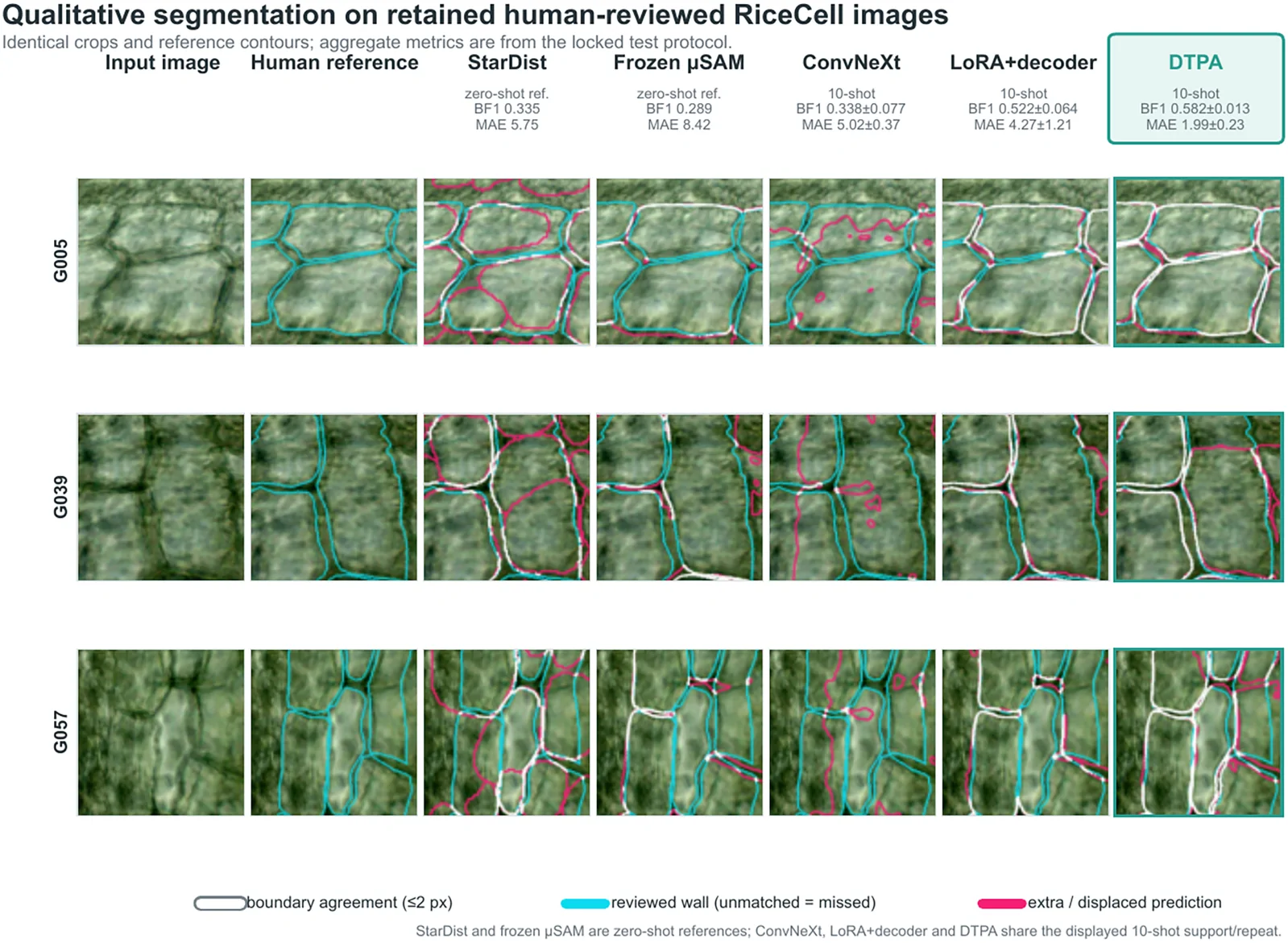
Qualitative boundary comparison on retained human-reviewed RiceCell images. Columns show identical crops from the raw image, human-reviewed reference, two zero-shot contextual references, and three 10-shot methods. Cyan marks the human-reviewed boundary; cyan without an overlaid prediction is therefore a missed boundary. White denotes agreement within two pixels, and magenta denotes an additional or displaced prediction. Header values are aggregate boundary F1 and cell-count MAE for the complete locked RiceCell test, not scores for the three crops. StarDist and frozen μSAM are zero-shot contextual references. ConvNeXt, LoRA+decoder AIS, and DTPA use the same support selection and repeat.

### 3.6 Domain-shift routing and locked external tests

The eight-dimensional domain-shift score separated the development domains. RiceCell support scores did not exceed 3.78, whereas the lowest PlantSeg validation score was 4.29. The retrospectively calculated potato median was 10.20. We fixed the threshold at 4.0 before opening the PlantSeg root test set. Dataset medians were 2.02 for RiceCell support, 2.77 for RiceCell validation, 5.58 for PlantSeg development, 5.71 for locked root, 6.39 for locked Ovules, and 7.68 for locked floral meristems (**Figure 7**). The router therefore selected the support-calibrated route for RiceCell and the query-adaptive route for every tested external collection at the frozen threshold.

**Figure 7:**
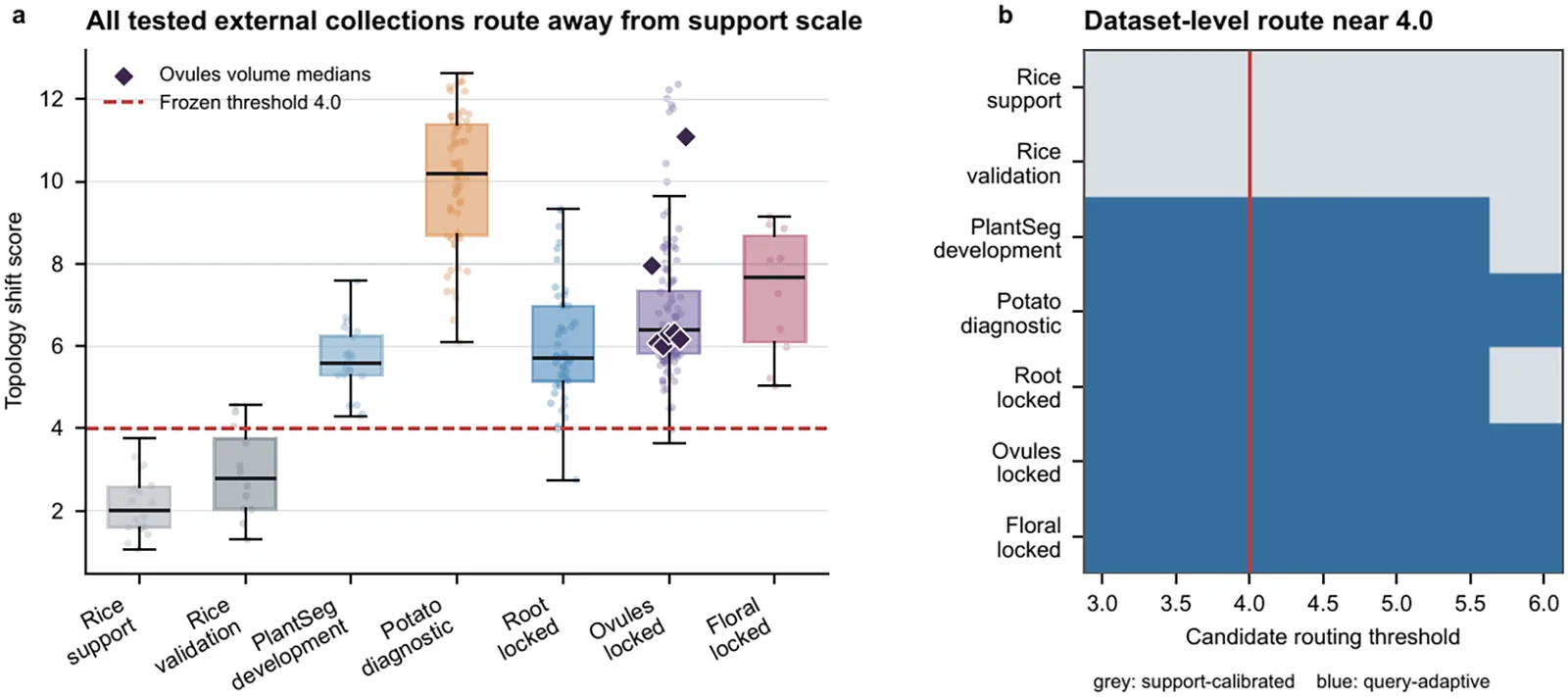
Domain-shift scores and retrospective routing sensitivity. **(a)** Per-image or per-slice descriptor-distance distributions for RiceCell, PlantSeg, potato, and floral-meristem data; the horizontal line is the frozen threshold of 4.0. **(b)** Fraction of observations assigned to the query-adaptive route as the candidate threshold varies from 2.5 to 8.0. The sweep reports routing coverage, not segmentation optimisation. Potato scores are retrospective diagnostics: the potato failure motivated the router concept but did not tune the descriptor or threshold.

In a retrospective threshold sweep from 3.0 to 6.0, RiceCell support and validation retained the support-calibrated route, whereas potato, Ovules, and floral meristems retained the query-adaptive route. PlantSeg development and locked root switched only when the candidate threshold exceeded their medians (5.58 and 5.71). The root decision is therefore stable near 4.0 but not across an arbitrarily broad threshold range. Ovules volume medians ranged from 6.00 to 11.09. This survey describes the available datasets and does not establish universal reliability of the routing rule.

Fixed support-scale prompting did not transfer to potato. On PlantSeg, the query-adaptive route increased SEG by 0.2017 and reduced cell-count MAE by 74.2% relative to the same frozen checkpoint (**Figure 8a**). Split and merge counts nevertheless increased. Routing thus reduced severe under-counting without improving every topology endpoint.

**Figure 8:**
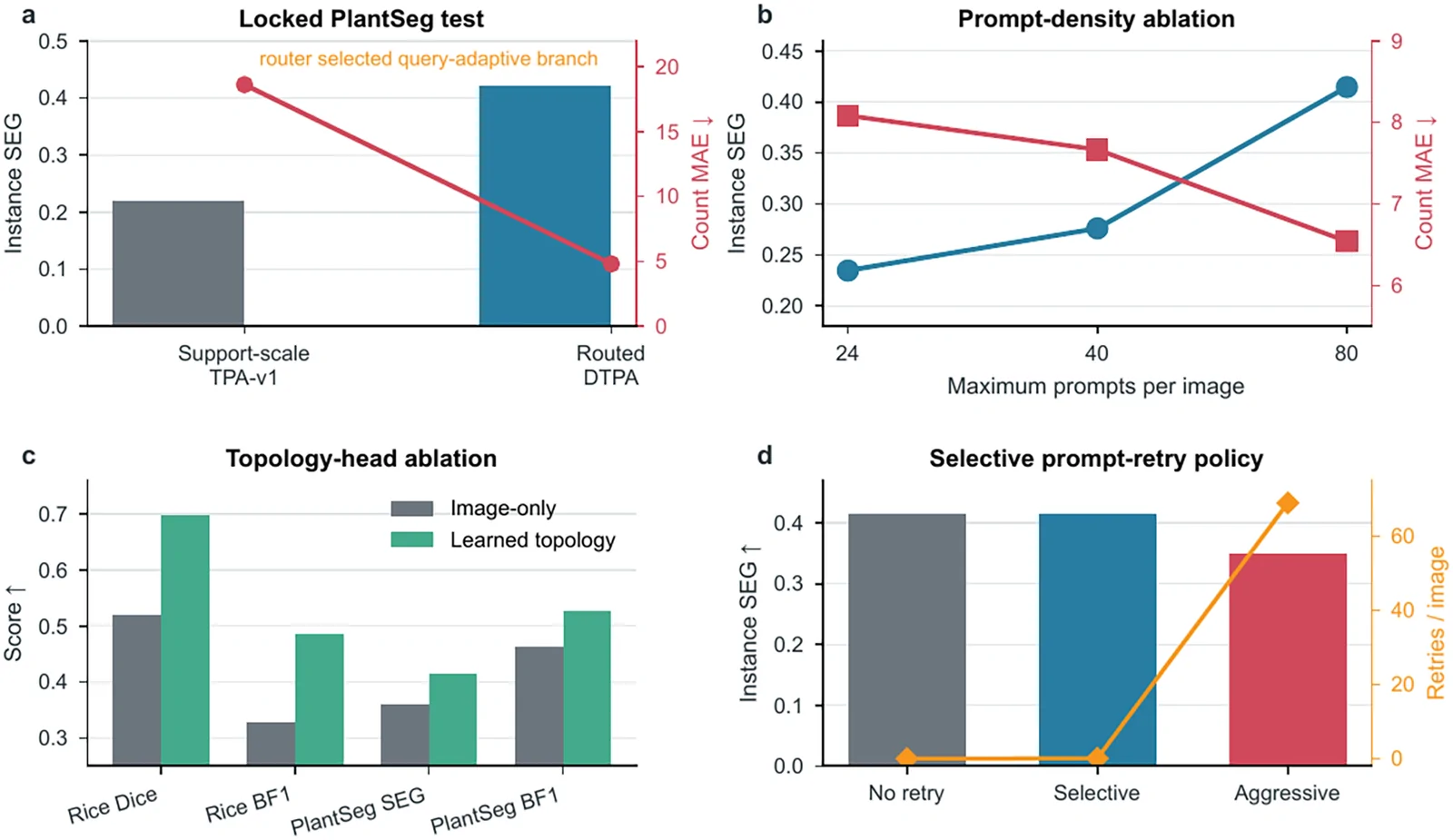
Locked external tests and component ablations. **(a)** On the single-pass locked PlantSeg test, the frozen domain-shift router selected the query-adaptive route. SEG increased, and cell-count MAE decreased relative to support-scale TPA-v1. **(b)** PlantSeg development performance as the prompt ceiling increased. **(c)** Region and boundary performance after replacing deterministic image edges with the topology adaptation head on RiceCell and PlantSeg validation data. **(d)** Selective retry remained close to no retry because the path-conflict gate rarely activated. Aggressive retry intervened more often and reduced SEG. **(b–d)** show development data only.

Ovules exposed a transfer boundary for the selective-correction controller (**Table 4**). The controller accepted 45 of 92 valid triggered events. The retrospective event oracle classified 22 as improvements, 14 as harms, and 9 as neutral. One additional trigger returned no valid retry mask and was excluded before scoring. The harm rate among accepted retries was 0.311, with an event-level Wilson 95% interval of 0.195–0.457. A 50,000-resample volume-cluster bootstrap gave an interval of 0.150–0.377; this interval remains imprecise because only seven volumes were available. Holm adjustment removed statistical significance from every paired volume-level endpoint.

**Table 4:** Locked PlantSeg Ovules test across seven independent three-dimensional volumes.

Metric	D	G	Δ [95% CI]	P_H
SEG ↑	.06283	.06283	-.000004 [-.000010,.000003]	1.000
Instance F1 (IoU 0.5) ↑	.09107	.09103	-.000042 [-.000070, -.000011]	1.000
Boundary F1 ↑	.21014	.21008	-.000055 [-.000123,.000002]	1.000
Cell-count MAE ↓	147.5616	147.5623	.0007 [-.0011,.0031]	1.000
Split/image ↓	1.2065	1.2009	-.0056 [-.0126,.0003]	1.000
Merge/image ↓	6.7884	6.7834	-.0050 [-.0111, -.0008]	1.000
Adjacency F1 ↑	.025832	.025837	.000005 [-.000017,.000025]	1.000
Normalised edge error ↓	3.1137	3.1133	-.000394 [-.001113, -.000020]	.344

Δ = G − D; intervals are bootstrap estimates of paired volume differences. Lower values are better for cell-count MAE, split, merge, and normalised edge error. P_H is Holm-adjusted across the prespecified 11-metric family. Eight metrics are shown; adjacency precision, adjacency recall, and degree MAE complete the multiplicity family and remain in the result archive.

The acceptance score did not reliably distinguish improvements from harms (**Figure 9**). The benefit-versus-harm AUROC was 0.594, with a volume-cluster bootstrap 95% interval of 0.505–0.714. The diagnostic ECE was 0.167, but this value is only a score–outcome discrepancy: the composite score was never trained or defined as a probability. Removing one component at a time retained 13–14 harms among 40–45 accepted retries (**Supplementary Table S5**). Across five area ranges, harm rates stayed between 0.308 and 0.314. Thresholds from 0.35 to 0.65 also retained 10–14 harms, with rates of 0.294–0.326. Internal conflict resolution was therefore not calibrated to reference-level benefit under this tested domain shift. **Supplementary Figure S8** reports the original score-ranked risk–coverage and reliability diagnostics.

**Figure 9:**
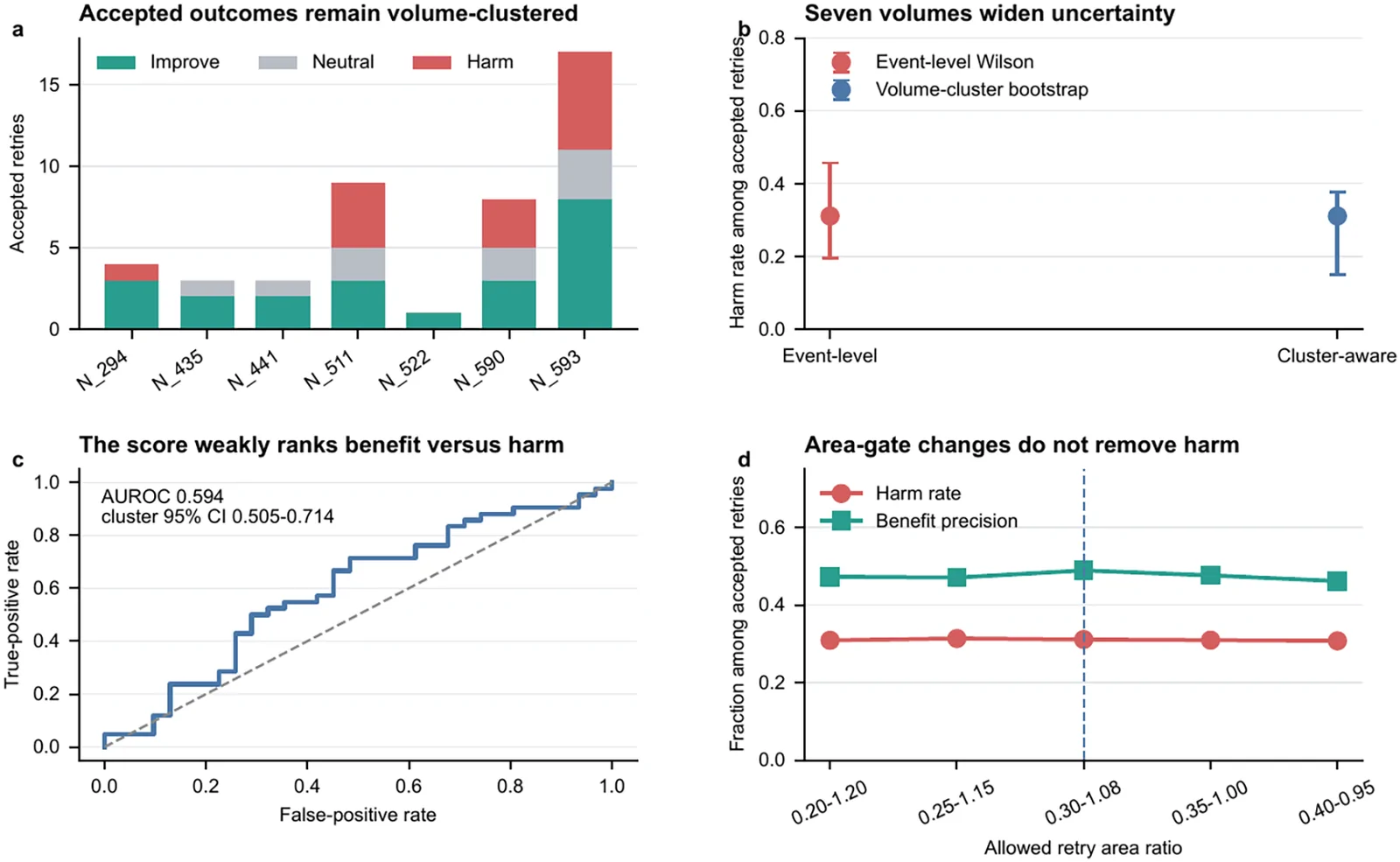
Cluster-aware uncertainty and controller sensitivity on the locked Ovules test. **(a)** Accepted improvement, neutral, and harm events within each of seven volumes. **(b)** Harm rate with an event-level Wilson interval and a volume-cluster bootstrap interval. **(c)** Receiver operating characteristic for benefit versus harm among non-neutral valid triggers; the interval resamples volumes. **(d)** Harm rate and benefit precision as the admissible retry-area range changes. The deployed range was 0.30–1.08. These retrospective analyses quantify uncertainty and rule dependence; they do not recalibrate the controller.

The event-oracle sensitivity analysis supported the same external-risk conclusion (**Supplementary Figure S1**). Across the core parameter grid, the number of harms ranged from 13 to 16 and the number of improvements from 22 to 24. Twelve of the 14 harms in the main setting persisted throughout the grid.

### 3.7 Independent cross-species stress test

The independently sourced floral-meristem data provided a stricter external test (**Supplementary Table S8**), in which instance F1 was 0.2250. Three events triggered the selective-correction controller. One was accepted and resulted in harm. Because the ten time points were nested within only two biological series, this observation documents a recurrence of the failure mode in the tested data but does not support population-level transfer. **Figure 10** summarises triggered and accepted correction outcomes across the validation, Ovules and independent floral-meristem tests.

**Figure 10:**
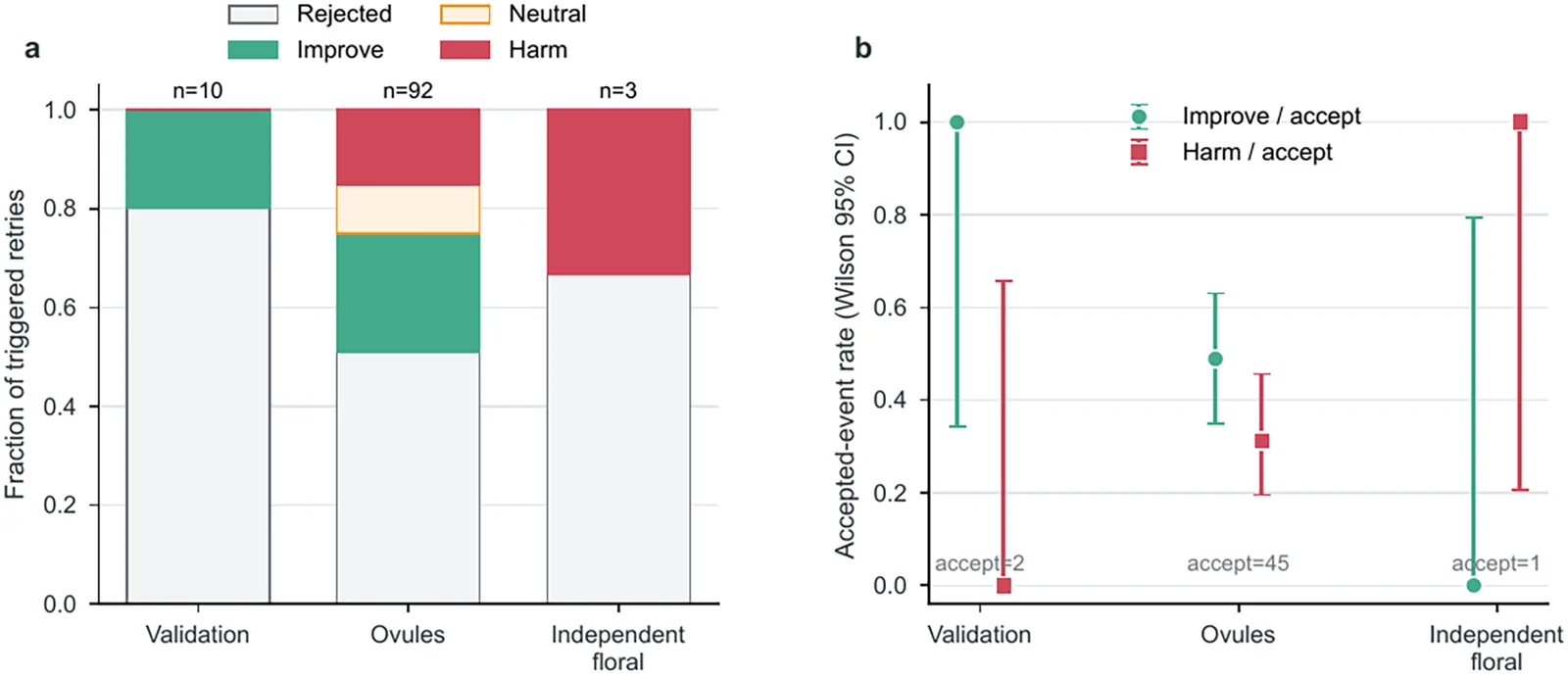
Selective-correction risk across evaluation domains. **(a)** Triggered events divided into rejections and accepted improvements, neutral outcomes, or harms. The controller accepted 2 of 10 validation events, 45 of 92 Ovules events, and 1 of 3 independent floral-meristem events. **(b)** Fractions of improvements and harms among accepted events, with Wilson 95% intervals. The floral-meristem interval is wide because only one event was accepted, and that event resulted in harm. Event counts, rather than slices, define the denominator.

### 3.8 Prompt budget and selective-retry ablation

Dense PlantSeg slices required broad prompt coverage. Raising the prompt ceiling from 24 to 80 increased SEG and reduced cell-count error (**Figure 8b**). Aggressive negative-prompt retry, however, intervened frequently and worsened both $F1_{0.5}$ and cell count. The selective-correction controller intervened infrequently (**Figure 8d**).

The factorial experiment separated these effects (**Supplementary Figure S2**; **Supplementary Table S1**). Prompt budget produced the clearest monotonic response. Learned-topology barriers gave the highest marginal SEG and the lowest cell-count error, whereas fused barriers gave the highest adjacency F1. Query-derived scale provided no independent benefit. Performance in the routed configuration therefore arose from an interaction among prompt coverage, barrier evidence, and scale, not from any one factor.

The post-review local sensitivity analysis reached the same bounded interpretation (**Supplementary Table S3**). The frozen 0.42 image weight, 0.76 barrier quantile, and 0.35 topology contrast gave SEG 0.4147 and boundary F1 0.5265. Changing one value at a time produced SEG values from 0.3863 to 0.4340 and boundary F1 values from 0.4488 to 0.5318. Some alternatives improved one endpoint while worsening another; no tested setting dominated the frozen configuration. These coefficients should therefore be treated as development-set heuristics rather than universal constants.

The staged A–G ablation supported the same interpretation (**Supplementary Table S6**). The topology adaptation head and query-adaptive route accounted for the majority of the change. By contrast, the selective-correction controller accepted only two validation events and altered one of 24 images. It should therefore be interpreted as a selective intervention mechanism with unresolved safety, not as an accuracy-improvement module.

### 3.9 Topology, redundancy, and robustness

Replacing learned topology probabilities with deterministic image edges reduced region and boundary performance (**Figure 8c**) without improving cell-count accuracy. This result is consistent with the topology adaptation head acting through the prompt field rather than directly correcting counts. Duplicate suppression was also domain-dependent. Disabling it had little effect on PlantSeg but increased RiceCell validation cell-count MAE from 3.58 to 22.92.

The corruption tests preserved this trade-off. DTPA changed little under Gaussian noise and only modestly under blur, but deteriorated most under low contrast. The complete metric grid is provided in the result files. DTPA trained 25,475 parameters, compared with 1.81 M for the ConvNeXt decoder and 9.74 M for LoRA+decoder AIS. The frozen backbone dominated computation, so the smaller trainable module did not shorten inference time.

## 4 Discussion

DTPA distinguishes two outcomes that are often treated as interchangeable: binary foreground overlap and the fidelity of cell-derived measurements. ConvNeXt achieved higher region overlap, whereas DTPA better preserved the designated boundary and cell-count endpoints. The human-reviewed audit extended this advantage to count, median cell area, and shape relative to ConvNeXt. Total projected-area differences were uncertain, and LoRA+decoder preserved adjacency degree better than DTPA. Boundary and count endpoints are especially relevant here because one missing wall can merge neighbouring cells without a proportionate loss of foreground pixels. They complement rather than supersede region overlap. The conclusion concerns a computational measurement tool, not a rice phenotype.

The topology adaptation head changes where and how prompts are placed while leaving the backbone fixed. This design differs from fully supervised boundary prediction and graph partitioning in PlantSeg (Wolny et al., 2020), flow-based instance reconstruction in Cellpose (Stringer et al., 2021), and star-convex object modelling in StarDist (Schmidt et al., 2018). DTPA instead learns from a small set of binary rice-cell masks where interior and boundary evidence should guide prompt placement. The equal-budget comparison with LoRA+decoder AIS indicates that this design trades some region overlap for better boundary and count fidelity. It does not outperform feature adaptation on every endpoint.

The ablations locate the main performance changes upstream of correction. The topology adaptation head changed the prompt field, dense prompt coverage was needed for crowded PlantSeg slices, and the router prevented indiscriminate reuse of RiceCell scale. By contrast, the controller altered few development images and retained substantial harm under the tested external shift. Prompt budget, topology-conditioned placement, and routing therefore carry the main positive evidence; correction remains a safety problem rather than a source of established accuracy gain.

The external tests exposed two transfer limits. Query-adaptive prompting reduced severe under-counting when pixel scale shifted, but split and merge errors moved in the opposite direction. Moreover, a plausible negative-prompt retry did not reliably predict reference-level benefit in shifted tissues. Routing and correction therefore require different operational responses. Domain shift can change the prompt route. The observed correction failures motivate disabling mask replacement and retaining $M_{1}$ in an uncalibrated shifted domain.

We derived the domain-level correction gate $g_{corr}$ from this post-audit failure analysis. The gate is a proposed deployment safeguard rather than a component of the tested pipeline. Prospective target-domain data are needed to determine whether it controls risk. The present results neither test the gate nor show that the acceptance score is calibrated.

## 5 Limitations

First, the RiceCell instances are candidate-assisted references. Within-reader repeatability was 6/12 ( κ=0.25). The two candidate-blinded readers agreed on 10/12 four-category decisions ( κ=0.75) and 11/12 retain/exclude decisions ( κ=0.80). Adjudication retained eight images and indicated that the connected-component proxy missed 5 of 73 instances. These results estimate proxy bias and review reproducibility; they are not independently acquired biological ground truth.

Second, the measurement analysis inherits the same reference limitation. Its eight images are audit cases rather than biological replicates, area remains in pixels, and the dataset contains neither treatment groups nor calibrated spatial units. The analysis tests whether segmentation preserves image-derived measurements; it cannot establish a developmental or physiological phenotype in rice. Count and area were stable across alternative references. Adjacency was reversed in the four-image exact-consensus subset, and the direction of the shape-factor result changed across reference definitions.

Third, evidence for generalisation is limited by 82 independent RiceCell families, two-dimensional evaluation of natively three-dimensional tissues, dataset-level routing, and synthetic corruption tests. The independent floral-meristem test contains ten time points from only two biological series. It strengthens the failure analysis but cannot establish population-level transfer. The 80-prompt route also required more computation and increased split and merge counts.

The baseline comparison has related constraints. Cellpose-SAM failed in the tested RiceCell configuration, and CellSAM weights were unavailable. The post-review target-trained StarDist comparison used all ten matched support selections but only one optimiser seed per selection; target-trained CellSAM and PlantSeg comparisons remain absent. The separate five-run fixed-support experiment describes optimiser-seed variation for DTPA, ConvNeXt, and LoRA+decoder, but not for StarDist. Neither repeated analysis expands the 12-family locked test set.

Finally, evidence for the selective-correction controller remains sparse. It accepted only two validation events, while the external audits contained harms that persisted across event-oracle settings. Neither development data nor external tests showed aggregate or adjacency-graph gains from accepting corrections. The proposed deployment risk budget and domain-level correction gate $g_{corr}$ were not evaluated in the reported pipeline and therefore require prospective validation.

## 6 Conclusion

DTPA uses topological evidence to adapt the prompt field of a frozen microscopy SAM backbone. On RiceCell, it improved the designated boundary and cell-count endpoints, although region overlap decreased and the instance references remained uncertain. Query-adaptive prompting reduced severe under-counting in a shifted tissue domain, whereas selective correction did not show acceptable risk in the tested shifted domains without target-domain calibration. The post-audit abstention rule remains an untested future safeguard. The contribution is therefore a bounded computational tool for plant-microscopy measurement fidelity. It does not establish a rice phenotype or universal transfer across plant tissues.

## Data Availability

The analysis, preprocessing and evaluation code, together with shareable reproducibility materials and analysis-ready derived data, are publicly available on Zenodo at https://doi.org/10.5281/zenodo.22746777 under the version identifier DTPA-RiceCell-RB-20260914-v1.2.1-public. The archive includes a file-level SHA-256 integrity manifest. Original RiceCell images and masks, derived prediction masks, model checkpoints and human-reviewed reference TIFFs are not publicly redistributed because access requires permission from the source-data provider. Requests for restricted RiceCell materials should be directed to the corresponding and data-contact author, Da Zeng (779102186@qq.com), who will coordinate permission with the provider. Public potato, PlantSeg and floral-meristem datasets are available from the sources cited in the Datasets subsection.
