# Supplementary material for: Topology-aware few-shot segmentation improves rice leaf-cell boundary and count fidelity, while correction is risky under tested domain shifts
Source: Front Plant Sci. 2026 Oct 13;17:1950986. doi: 10.3389/fpls.2026.1950986 (PMC13710281; doi:10.3389/fpls.2026.1950986)
Supplement: Supplementary file 1 [file DataSheet1.pdf]

Supplementary Material

This file contains the supplementary figures and tables cited in the main manuscript. In accordance with the Frontiers guidelines for supplementary material, it does not repeat the title block, author list, affiliations or correspondence details.

Supplementary Fig. S1: event-oracle sensitivity

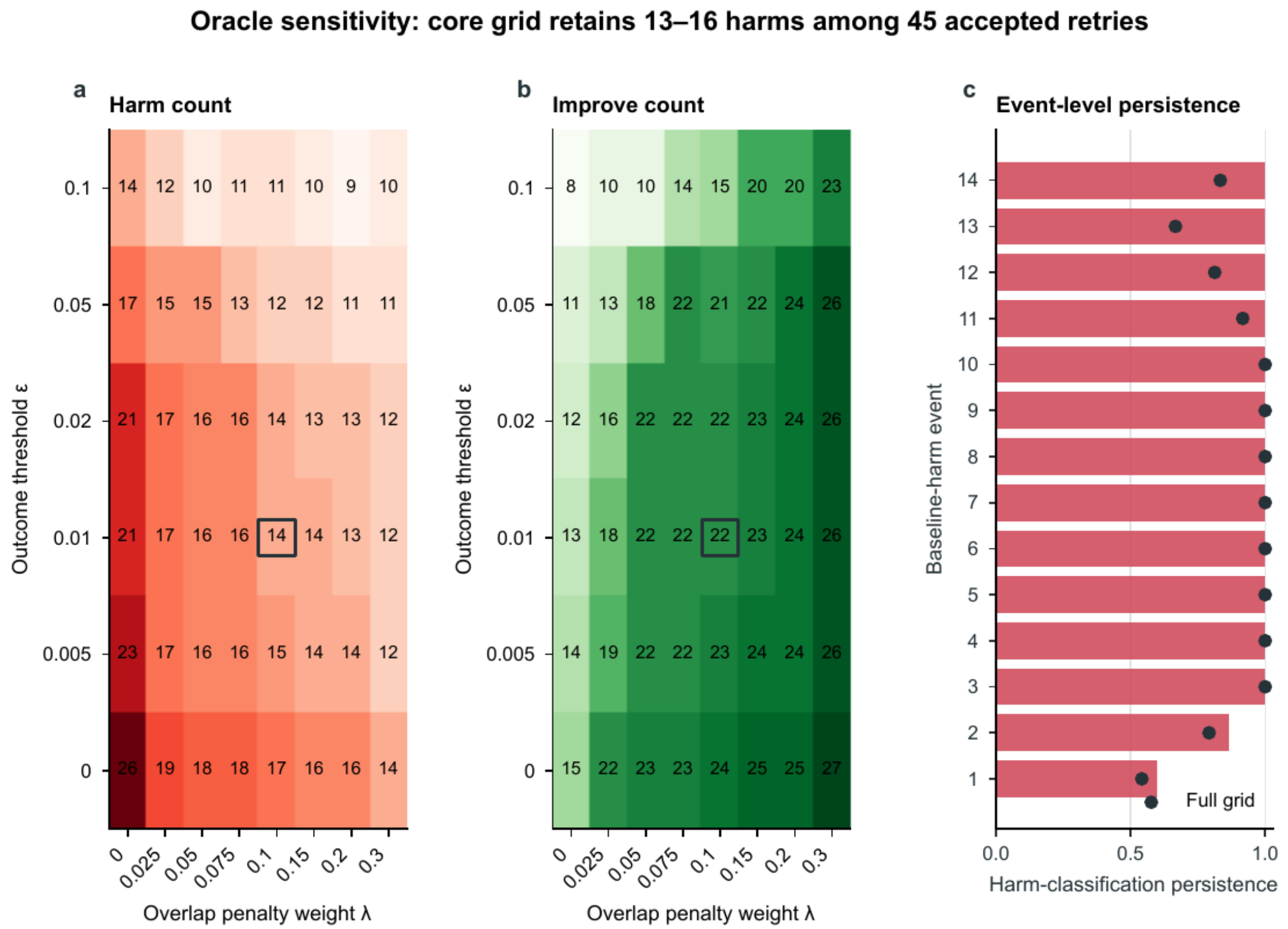

Sensitivity of the retrospective event oracle for 45 accepted Ovules retries. Counts of improvements and harms are shown as the overlap-degree weight λ and outcome threshold ε vary. The outlined cell indicates the main setting, $(0.10, 0.01)$*. The persistence panel shows the fraction of core-grid settings in which each event classified as a harm in the main setting remains a harm. The core grid spans* $\lambda = 0.05$*–0.20 and* $\varepsilon = 0.005$*–0.02. Twelve of the 14 harms persist throughout the grid. These retrospective labels were not used to train or tune the acceptance gate.*

Supplementary Fig. S2: prompt-construction factors

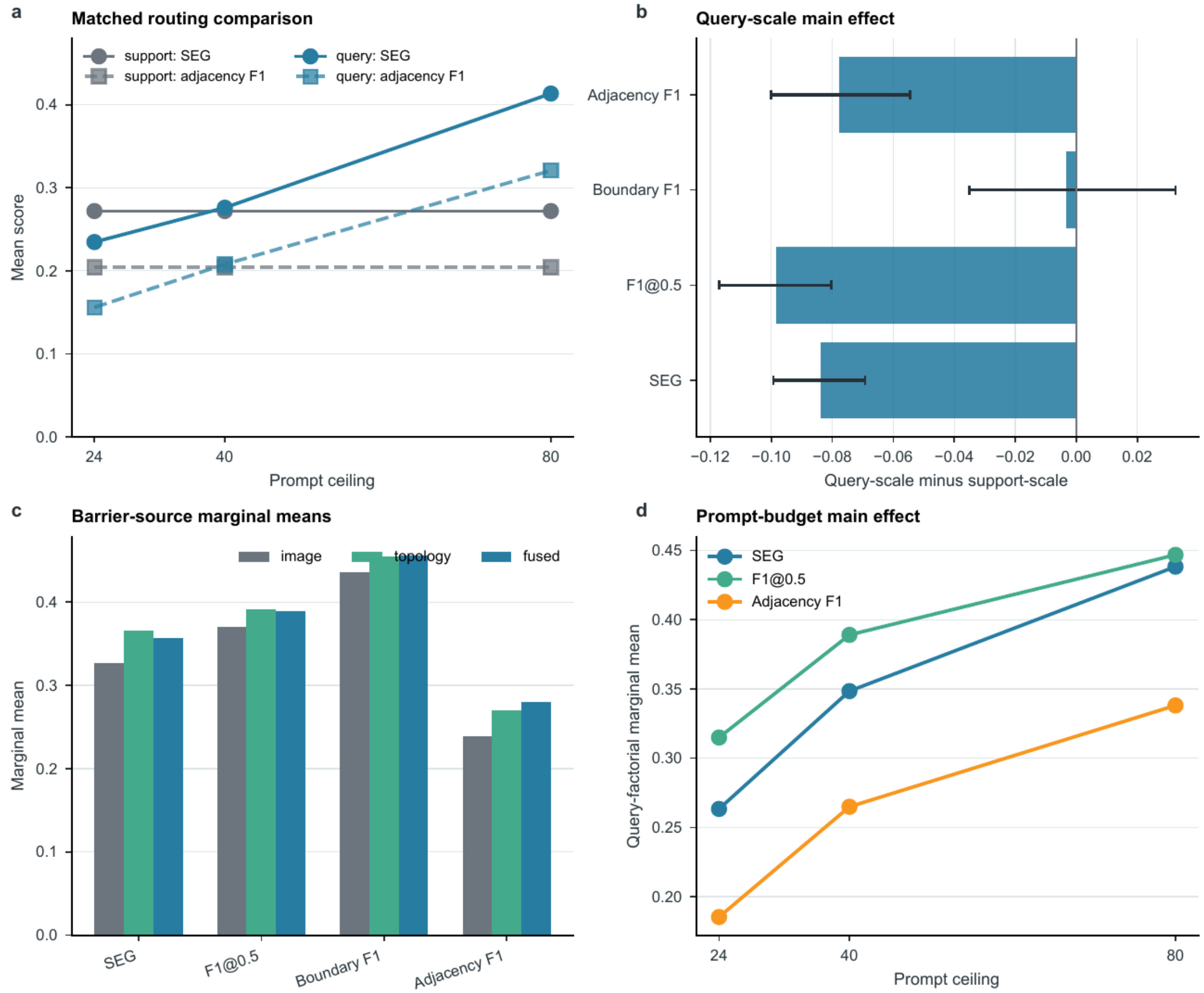

Factorised prompt-construction ablation using 24 PlantSeg validation slices. The analysis compares matched support-calibrated and query-adaptive routes across prompt ceilings. It also reports query-minus-support scale effects with image-level bootstrap 95% intervals, together with marginal means for barrier source and prompt budget. We disabled negative-prompt retry and kept duplicate suppression fixed.

Supplementary Table S1: factorised prompt ablation

Separate routing, query-scale, barrier-source and prompt-budget factors on PlantSeg validation data. The scale, barrier and budget rows show query-factorial marginal means, while the route rows show matched 80-prompt configurations.

Factor	Level	SEG	$F1_{0.5}$	Boundary F1	Cell-count MAE	Adj. F1
Route	Support calibrated (80)	0.2719	0.4260	0.7292	10.71	0.2041
Route	Query adaptive (80)	0.4135	0.4202	0.5268	6.46	0.3207
Scale	Support	0.3919	0.4328	0.4507	7.00	0.3017
Scale	Query	0.3081	0.3344	0.4473	7.60	0.2238
Barrier	Image	0.3272	0.3701	0.4361	7.56	0.2384
Barrier	Topology	0.3660	0.3919	0.4552	7.12	0.2699
Barrier	Fused	0.3569	0.3887	0.4556	7.22	0.2799
Budget	24	0.2633	0.3149	0.4020	8.33	0.1853
Budget	40	0.3484	0.3890	0.4517	6.90	0.2649
Budget	80	0.4383	0.4468	0.4933	6.67	0.3381

Supplementary Table S2: candidate-assisted reader agreement

Agreement in the candidate-assisted review of 12 locked RiceCell images. Brackets give image-level bootstrap 95% intervals for Cohen's κ. This audit is secondary to the matched binary-mask endpoints.

Comparison	Exact agreement	Cohen's kappa
Same reader, four-category	6/12 (50.0%)	0.25
Two readers, four-category	10/12 (83.3%)	0.75 [0.39, 1.00]
Two readers, retain/exclude	11/12 (91.7%)	0.80 [0.23, 1.00]

Supplementary Table S3: prompt-parameter sensitivity

Post-review one-factor-at-a-time sensitivity analysis on 24 PlantSeg development slices. Correction was disabled and duplicate suppression was fixed. The first row is the frozen configuration used for external tests.

Configuration	SEG	$F1_{0.5}$	Boundary F1	Count MAE	Adj. F1
Frozen: $w_{img}=.42, q=.76, c=.35$	.4147	.4207	.5265	6.54	.3192
$w_{img}=.25$	.4336	.4275	.5034	7.04	.3288
$w_{img}=.60$	.3974	.4125	.4853	6.46	.3065
Barrier quantile $q=.70$	.3863	.3654	.4488	10.08	.2811
Barrier quantile $q=.82$	.4340	.4404	.5318	6.46	.3613
Topology contrast $c=.25$	.4175	.4347	.4904	6.17	.3360
Topology contrast $c=.45$	.4201	.4270	.5026	6.67	.3259

Supplementary Table S4: matched 10-shot measurement analysis

For each method and matched 10-shot support selection, measurement errors were first averaged across the same eight retained human-reviewed RiceCell images. The table then averages the ten support-selection summaries. The ten matched support selections, not the eight audit images, were the bootstrap units for the reported matched-analysis intervals. Lower values are better. The projected-area row measures total foreground area; the median-area row measures the typical connected instance.

Endpoint	DTPA	ConvNeXt	LoRA+decoder
Cell-count absolute error	.900	6.600	2.263
Projected-area relative error	.112	.137	.143
Median cell-area relative error	.205	4.793	.301
Median shape-factor absolute error	.057	.244	.077
Mean adjacency-degree absolute error	.699	2.492	.551

Paired bootstrap intervals for DTPA minus ConvNeXt excluded zero for count, median cell area, shape factor and adjacency degree, but included zero for projected area. Against LoRA+decoder, intervals excluded zero for count and adjacency degree; intervals for projected area, median cell

area and shape factor included or approached zero. The adjacency direction favoured LoRA+decoder over DTPA. These are secondary support-selection sensitivities, not population estimates.

Supplementary Table S5: controller-rule sensitivity

Retrospective sensitivity of accepted Ovules correction events. The full controller used score threshold 0.45 and area ratio 0.30–1.08. Removing a score term renormalised the remaining weights. These analyses diagnose the original event record and do not retune the controller.

Rule	Accepted	Improve	Harm	Neutral	Harm rate
Full controller	45	22	14	9	.311
Without conflict-removal term	40	19	13	8	.325
Without path-support term	44	22	14	8	.318
Without decoder-retention term	45	22	14	9	.311
Without topology-consistency term	45	22	14	9	.311
Without area-plausibility term	44	22	14	8	.318
Area ratio .20–1.20	55	26	17	12	.309
Area ratio .40–.95	39	18	12	9	.308
Score threshold .35	45	22	14	9	.311
Score threshold .65	34	18	10	6	.294

Supplementary Table S6: A–G module ablation

A–G module ablation on PlantSeg validation data. Adjacency F1 evaluates the matched cell-neighbourhood graph. Event counts are totals across 24 slices.

Cfg.	Added component	SEG	$F1_{0.5}$	Boundary F1	Cell-count MAE	Split	Merge	Adj. F1	Trigger/ accept/ improvement/harm
A	Image-only support prompts	.0436	.0821	.1899	14.29	.00	.21	.0000	0/0/0/0
B	Topology adaptation head	.2754	.4315	.7334	10.88	.38	1.29	.2011	0/0/0/0
C	Query-adaptive scale and barrier paths	.4172	.4231	.5275	6.63	1.75	1.96	.3221	0/0/0/0

Cfg.	Added component	SEG	$F1_{0.5}$	Boundary F1	Cell-count MAE	Split	Merge	Adj. F1	Trigger/ accept/ improvement/harm
D	Duplicate suppression	.4147	.4207	.5265	6.54	1.63	1.92	.3192	0/0/0/0
E	Legacy overlap-based retry	.4147	.4207	.5265	6.54	1.63	1.92	.3192	2/0/0/0
F	Topology path-conflict gate	.4147	.4207	.5265	6.54	1.63	1.92	.3192	10/1/1/0
G	Correction acceptance score	.4146	.4210	.5268	6.58	1.58	1.88	.3188	10/2/2/0

Supplementary Table S7: audit-reference sensitivity

Sensitivity of the RiceCell audit to the candidate-assisted reference selection. Each cell reports DTPA/StarDist. The primary column uses the eight human-reviewed references. The range covers all 16 proxy/prelabel combinations for G033, G039, G064 and G072, the retained images for which the second reader and final adjudication differed. The second-reader scenario also retains G027 ($n = 9$); the consensus scenario includes only the four retained images for which both readers selected the same candidate.

Endpoint	Primary $n = 8$	All legal $n = 8$ choices	Second reader $n = 9$	Reader consensus $n = 4$
SEG ↑	.6402/.5251	.6402–.6468/.5251–.5277	.6092/.4850	.7590/.6062
$F1_{0.5}$ ↑	.7443/.4035	.7443–.7716/.4035–.4082	.7018/.3629	.8523/.4466
Boundary F1 ↑	.6354/.3610	.6354–.6354/.3610–.3610	.6189/.3603	.6890/.3631
Cell-count MAE ↓	.625/6.500	.625–.875/6.250–6.500	1.000/5.556	.500/7.500
Median-area error ↓	.2889/.3902	.2889–.3398/.3672–.3902	.3766/.3961	.0932/.4644
Shape-factor error ↓	.0567/.0551	.0549–.0567/.0532–.0551	.0731/.0651	.0338/.0791
Adjacency-degree error ↓	.6031/.9625	.5990–.6031/.9584–.9625	.6435/.9260	.6333/.5085

DTPA had higher SEG, $F1_{0.5}$ and boundary F1, and lower cell-count and median-area errors, under all 16 $n = 8$ candidate combinations and the second-reader $n = 9$ scenario. The direction of the shape-factor result changed across reference definitions. Adjacency-degree error was lower for

DTPA in all 16 $n=8$ combinations and the $n=9$ scenario, but the direction reversed in the four-image consensus subset. These are descriptive reference-sensitivity analyses, not alternative ground truths.

Supplementary Table S8: independent floral-meristem stress test

Locked Arabidopsis floral-meristem stress test. The ten median slices are nested within two biological series. Values are descriptive, with $\Delta = G - D$ *. This table serves as a failure-boundary analysis and does not provide evidence of population-level transfer.*

Metric	D	G	Δ
SEG ↑	.138235	.138229	-.000006
$F1_{0.5}$ ↑	.225045	.225045	.000000
Boundary F1 ↑	.485492	.485200	-.000291
Cell-count MAE ↓	129.70	129.70	.00
Merge errors ↓	13.40	13.40	.00
Adjacency F1 ↑	.093162	.093162	.000000
Retry events	trigger 3 / accept 1 / improvement 0 / harm 1		

Supplementary Table S9: fixed-support optimiser-seed replication

Five optimiser-seed runs per method with the same repeat-0 10-shot support families. Values are mean ± sample standard deviation [minimum, maximum]. The test set remained the same 12 locked families.

Method	Dice	Boundary F1	Cell-count MAE
DTPA	.8324 ± .0048 [.8252,.8375]	.5981 ± .0053 [.5952,.6075]	2.30 ± .07 [2.25,2.42]
ConvNeXt	.8665 ± .0029 [.8617,.8684]	.2957 ± .0320 [.2436,.3248]	4.97 ± .17 [4.67,5.08]
LoRA+decoder	.8439 ± .0125 [.8287,.8562]	.4825 ± .0753 [.3850,.5388]	4.13 ± .69 [3.42,5.17]

Supplementary Table S10: target-trained StarDist matched-support baseline

Post-review StarDist specialist baseline trained on the same ten 10-shot support selections used by DTPA and LoRA+decoder. Each row uses one registered optimiser seed. Probability and non-maximum-suppression thresholds were selected on the fixed validation families before evaluation on the same 12 locked test families.

Support repeat	Dice	Boundary F1	SEG	$F1_{0.5}$	Count MAE
0	.425	.282	.246	.296	3.83
1	.661	.357	.378	.429	3.42
2	.659	.381	.382	.426	2.58
3	.691	.390	.491	.421	3.08
4	.686	.385	.400	.418	2.67
5	.748	.412	.474	.514	2.92
6	.727	.411	.495	.493	2.50
7	.715	.394	.470	.488	2.25
8	.701	.411	.479	.461	2.67
9	.609	.371	.342	.411	3.08
Mean $\pm$ SD	$.662 \pm .092$	$.379 \pm .039$	$.416 \pm .081$	$.436 \pm .061$	$2.90 \pm .47$

Supplementary Fig. S3: visual audit-reference sensitivity

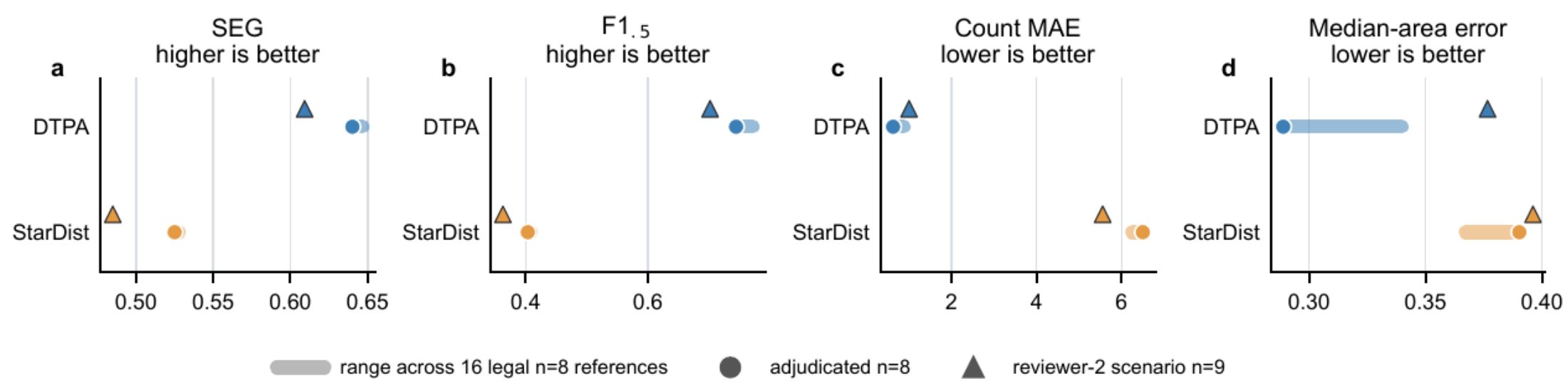

Reference-selection sensitivity for four representative audit endpoints. Thick lines span all 16 admissible proxy/prelabel combinations for the same eight retained images. Circles mark the human-reviewed reference, and triangles mark the second-reader scenario that additionally retains G027. The ranges describe reference sensitivity rather than confidence intervals.

Supplementary Fig. S4: complete retained audit gallery, part A

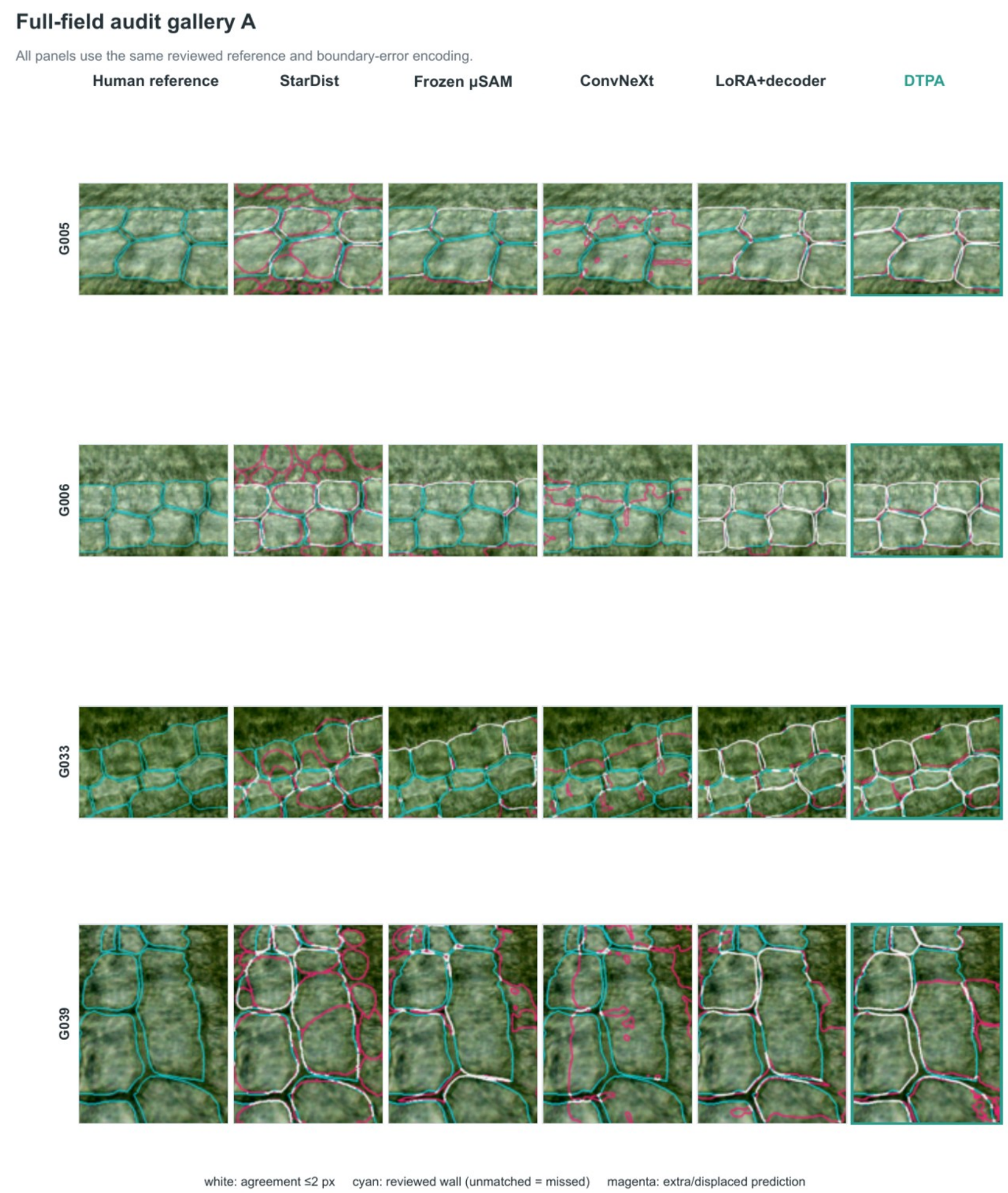

Supplementary Figure S4. Full-field boundary-error overlays for the first four retained human-reviewed RiceCell images. White denotes prediction–reference agreement within two pixels, cyan denotes a missed human-reviewed boundary, and magenta denotes an additional or displaced prediction. StarDist and frozen μSAM are zero-shot contextual references. ConvNeXt, LoRA+decoder AIS and DTPA use the same displayed 10-shot support selection and repeat.

Supplementary Fig. S5: complete retained audit gallery, part B

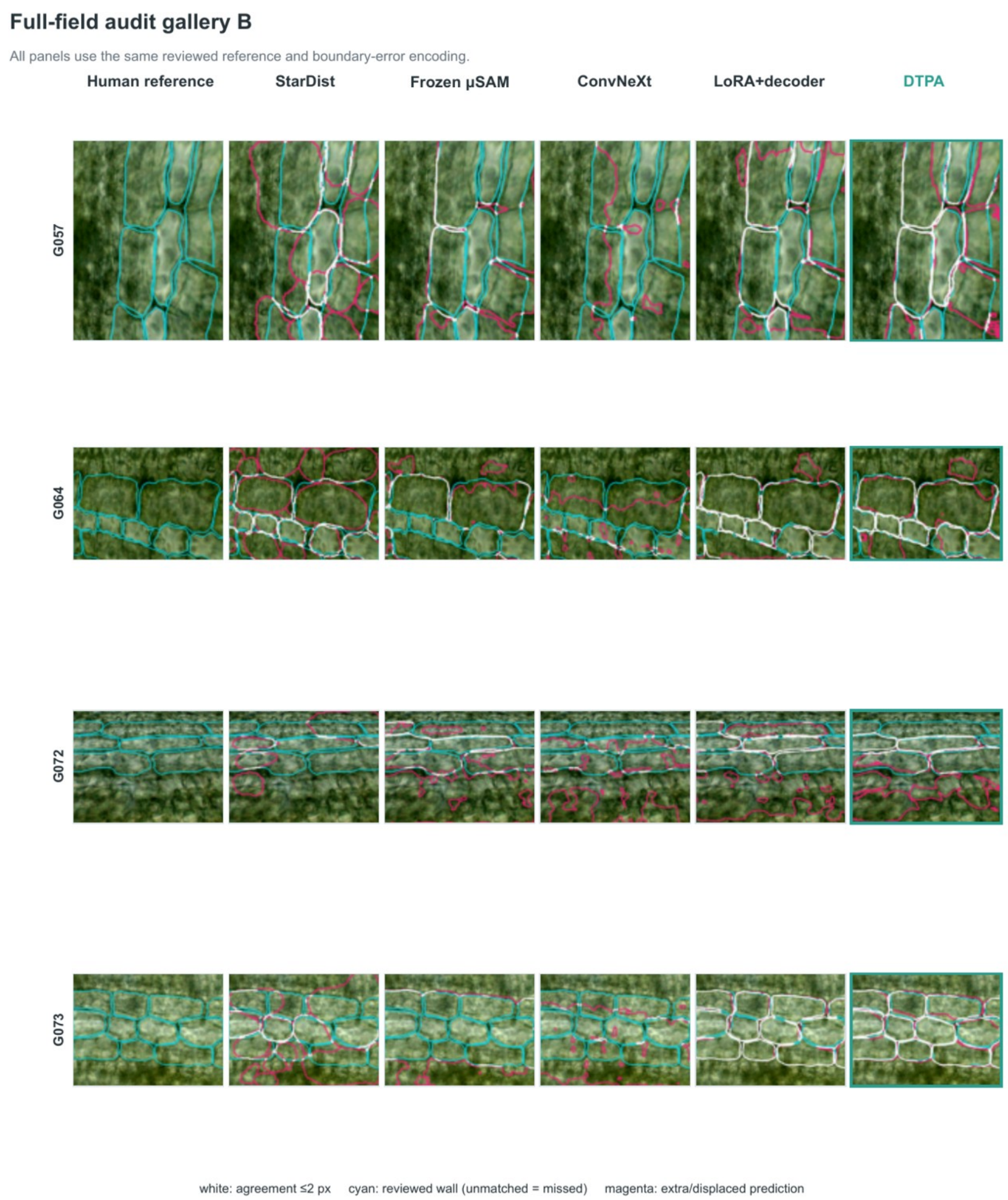

Supplementary Figure S5. Full-field boundary-error overlays for the remaining four retained human-reviewed RiceCell images, using the same methods, reference masks and colour encoding as Supplementary Fig. S4. Together, Supplementary Figs. S4–S5 present all eight retained audit images rather than only the three crops highlighted in the main text.

Supplementary Fig. S6: connected-component proxy audit

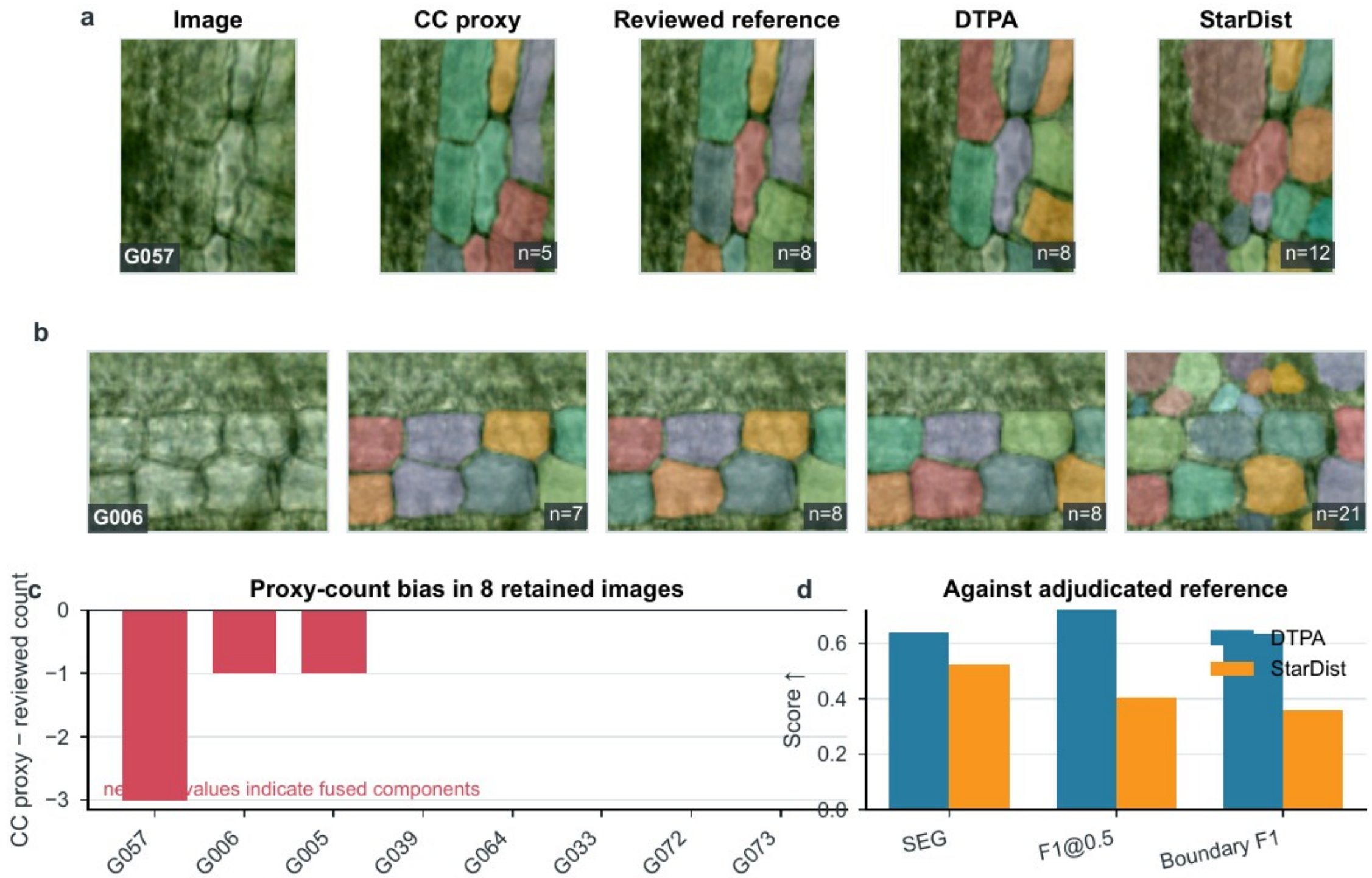

Candidate-assisted review of connected-component proxy instances. Panels a–b show two retained images with the proxy, human-reviewed reference, 20-shot DTPA and zero-shot StarDist; n *is the instance count. Panel c shows the proxy-minus-reviewed count difference, and panel d gives segmentation metrics for the eight retained images. This 20-shot reference-bias audit is separate from the matched 10-shot analysis.*

Supplementary Fig. S7: secondary DTPA–StarDist morphometric audit

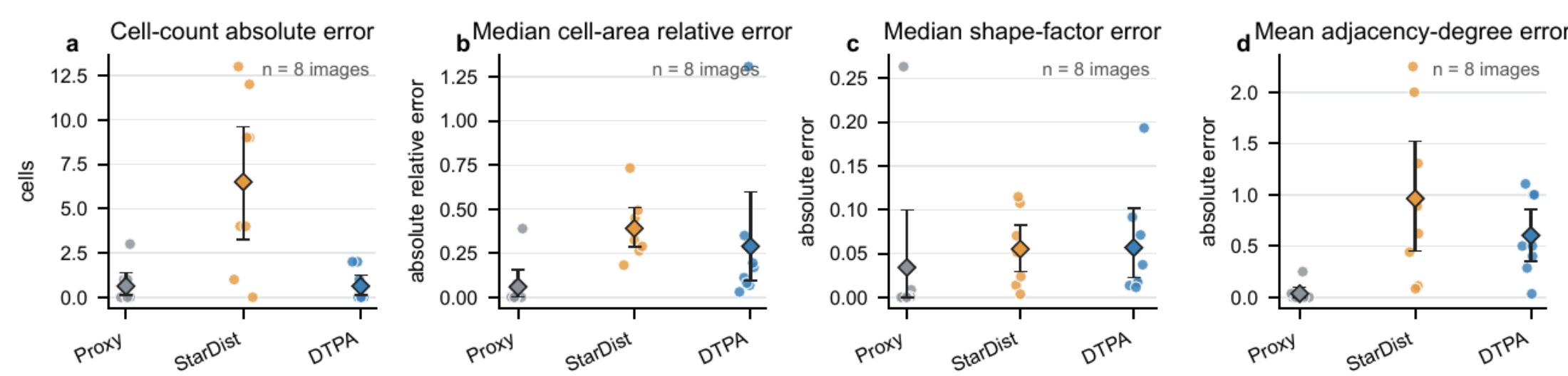

Pixel-scale measurement errors in eight retained, human-reviewed RiceCell images for the pre-specified 20-shot repeat-0 DTPA prediction, zero-shot StarDist and the connected-component proxy. Panels show absolute cell-count error, median cell-area relative error, median shape-factor

error and mean adjacency-degree error. Points are images; diamonds and bars are means and image-level bootstrap 95% intervals. The images are audit cases, not biological replicates.

Supplementary Fig. S8: score-ranked correction diagnostics

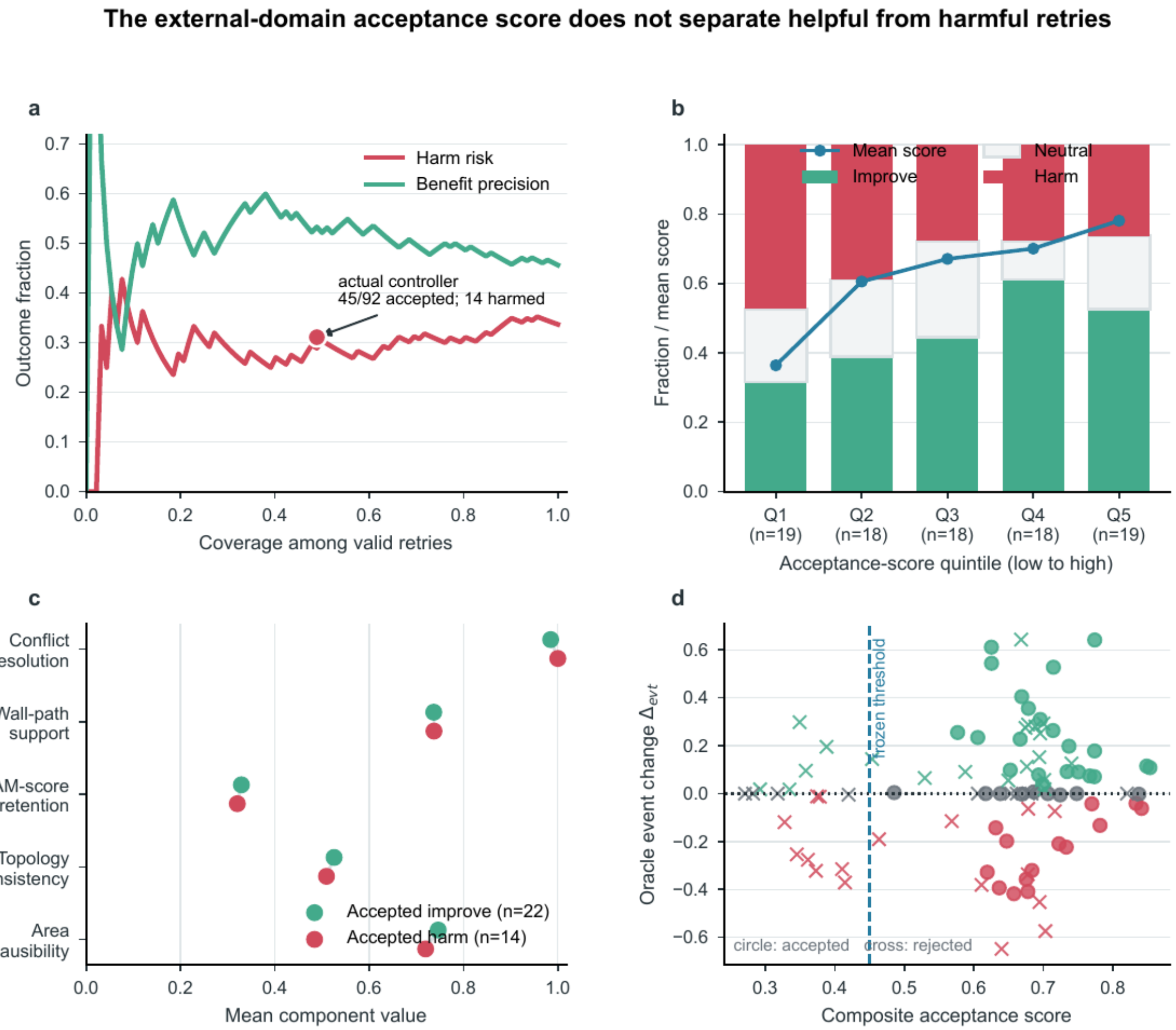

Risk–coverage and reliability diagnostics for 92 valid triggered retries on the locked Ovules test. The all-trigger ledger contains 93 events; one event returned no finite, non-empty retry candidate and was excluded before acceptance scoring and oracle classification. Panels show score-ranked harm risk and benefit precision, equal-frequency score bins, score-component means and the composite score against the retrospective

event oracle. The score is a hand-specified composite gate, not a learned probability; the reported ECE is therefore diagnostic rather than a calibration estimate.

Supplementary Fig. S9: fixed-support optimiser-seed replication

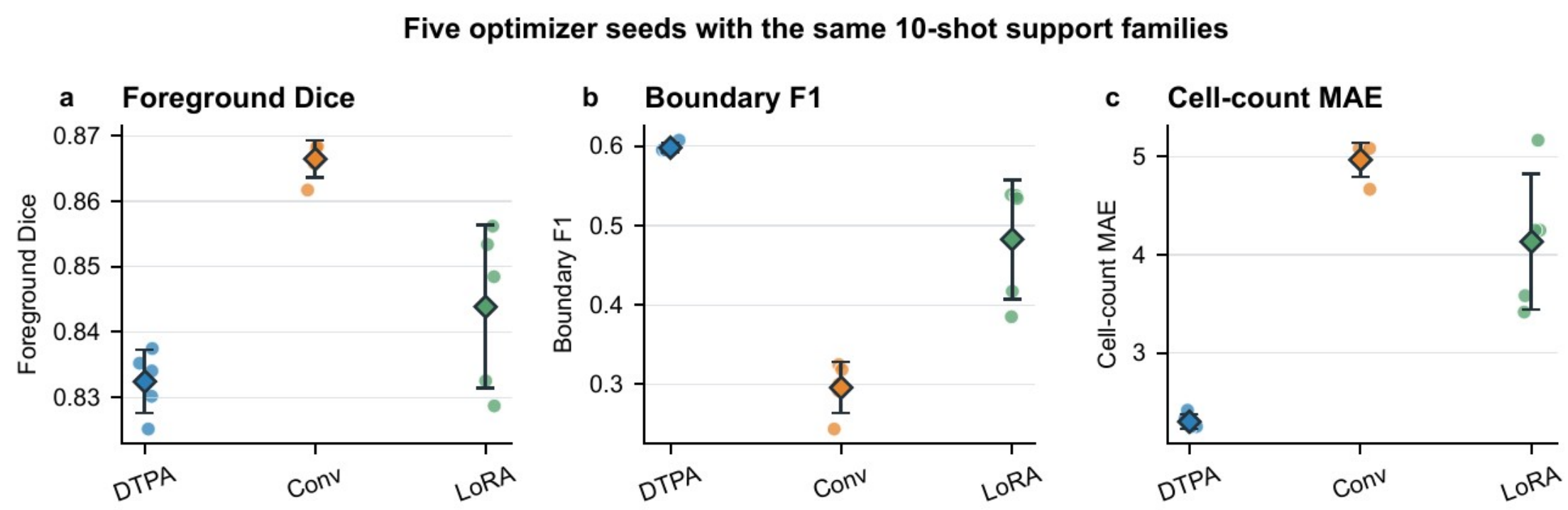

Foreground Dice, boundary F1 and cell-count MAE across five optimiser-seed runs for DTPA, ConvNeXt and LoRA+decoder. Every run used the same repeat-0 10-shot support identities and the same 12 locked test families. Points are runs; diamonds and bars show the mean and sample standard deviation. This experiment isolates training stochasticity for one support selection.
