# Supplementary material for: Topology-aware few-shot segmentation improves rice leaf-cell boundary and count fidelity, while correction is risky under tested domain shifts
Source: Front Plant Sci. 2026 Oct 13;17:1950986. doi: 10.3389/fpls.2026.1950986 (PMC13710281; doi:10.3389/fpls.2026.1950986)
Supplement: Supplementary file 2 [file DataSheet2.pdf]

Independent Statistical Reverification Statement

Manuscript 1950986   Author correction packet dated 9 September 2026

Computational conclusion   Statistically verified; declaration executed electronically

This statement records the outcome of an independent statistical re-verification of the frozen correction packet. All four material issues identified in the first review were resolved. The statistician authorised use of the electronic signature shown in this declaration, as confirmed by the authors.

Conclusion

The corrected statistical results are verified within the stated review scope. Forty-six of 49 prespecified results matched the author values within the numerical tolerance. Three confidence-interval endpoints showed acceptable Monte Carlo variation under an independent random stream, with no change in any point estimate, direction, significance decision or interpretation. No material numerical discrepancy remains.

Scope and methods

Review component	Completed work
Input integrity	99 of 99 PACKET SHA-256 entries and 32 of 32 source-freeze entries verified; no package-manifest mismatch.
Independent computation	Analysis units, pairing, bootstrap intervals, exact Wilcoxon tests, Holm correction, Wilson interval, volume-cluster bootstrap, AUROC, ECE, seed metadata and reader agreement were recomputed without importing author result scripts.
Author cross-check	Author scripts were run only after independent outputs were locked. Their statistical outputs agreed with the frozen author tables.
Document review	The 29-page revised manuscript, 13-page supplement and two-page correction response were rendered and checked for analysis-unit and conclusion-boundary consistency.

Material issue resolution

Issue	Subject	Status	Reverification finding
IR-001	Canonical boundary stream	Reverified	ConvNeXt 10-shot boundary F1 and paired statistics now use one traceable native-grid per-image stream throughout the manuscript and outputs.
IR-002	Complete trigger ledger	Reverified	All 93 triggers were reconstructed from three raw JSON shards, including the single event without a valid retry.
IR-003	Seeds and RNG metadata	Reverified	The 86-row registry contains numeric training and bootstrap seeds, versions, resample counts, stream rules and undefined-sample handling.

Issue	Subject	Status	Reverification finding
IR-004	Analysis-unit wording	Reverified	The manuscript now separates the eight-image descriptive audit from the ten-support matched bootstrap unit.

Key numerical results

Endpoint	Independent result	Interpretation
ConvNeXt boundary F1	0.324231198535	Sample SD 0.077120921140; ten matched support selections
DTPA minus ConvNeXt	0.257973432606	Paired bootstrap 95% CI 0.209435866682 to 0.306010949321
Wilcoxon and Holm	0.001953125; 0.005859375	Exact two-sided p; Holm-adjusted p across three prespecified endpoints
Controller ledger	93 total; 92 valid; 1 invalid	45 accepted: 22 improve, 14 harm, 9 neutral
Accepted-event harm	0.311111111111	Wilson 95% CI 0.195286992637 to 0.456648055774
Cluster harm interval	0.150000000000 to 0.377358490566	50,000 bootstrap samples of seven Ovules volumes
Benefit-vs-harm AUROC	0.594470046083	Volume-cluster 95% CI 0.505116279070 to 0.714315476190
ECE	0.166710808288	Diagnostic only; acceptance score is not a calibrated probability

Interpretation boundaries

The manuscript limits the matched-domain claim to the locked RiceCell families and support selections. The eight reviewed images are candidate-assisted descriptive audit cases, not biological replicates. Ovules uncertainty is clustered by seven volumes and remains wide. The floral-meristem evidence comprises two biological series. Pixel-scale measurements have no physical calibration and do not support a rice phenotype, universal transfer claim or biological mechanism. Controller harms remain present under the tested domain shifts, and the proposed deployment gate has not been prospectively validated.

Scope qualification

The frozen table-level material is sufficient for the mandatory statistical re-verification. A fully portable package-only pixel-to-result run is not claimed: several author scripts and seed-source references retain absolute author-machine paths, and restricted images, predictions, reference labels and checkpoints require controlled access. This does not change the verified statistical results because pixel-to-result rerunning was a conditional scope item in the review protocol. Package-relative paths and source hashes are recommended for long-term archival portability.

Verified files

Artifact	SHA-256
Author correction ZIP	FDAE3438D7AA27250BEF750E2B225B0574A589AB2DA4996 6BF0BEE5014D7037A
Revised manuscript clean PDF	76103B5A32A209AC966ECAAC46325A6E3FAA3E4517B336C E4D6A9BA193C87441
Independent output manifest	DB811C10C8DCA2671BD9D2016512C849D0717940D33DBA 8D61AA3B12696F4C9B
Completed review workbook	F1334BB09C27624BCF42B174E48CF49F1D461BDF571EA95 F04DF73CFD5700D95

Reviewer declaration and signature

EXECUTED ELECTRONIC DECLARATION — the signature below was supplied for this statement and used with the statistician's authorisation, as confirmed by the authors.

Executed declaration by the independent statistician: I confirm that I did not participate in the original statistical analysis or result selection, except as disclosed below. I reviewed the frozen materials and the independent outputs described in this statement. Subject to the scope and limitations stated above, I conclude that the corrected statistical results and manuscript claims are verified.

Field	Reviewer entry
Full name and degree	Wenjing Li; Bachelor of Science in Mathematics; postgraduate education
Position and institution	Second-level Professor, Nanning Normal University
Professional email	Professional email: liwj@nnnu.edu.cn<br>Alternative contact email: 1971878871@qq.com
Relevant statistical experience	Professor specializing in parallel and intelligent computing; principal investigator of three National Natural Science Foundation of China projects and more than ten major or regional science and technology projects; author of more than 100 academic papers, including over 30 SCI/EI-indexed papers. Relevant experience includes computational reproducibility, numerical implementation and independent verification of machine-learning analyses.
Independent of original analysis and result selection	[X] Yes [ ] No If no, explain:
Conflict of interest	[X] None [ ] Disclosed below
Disclosure if applicable	Not applicable

Field	Reviewer entry
Signature	Electronic signature (used with the statistician's authorisation): 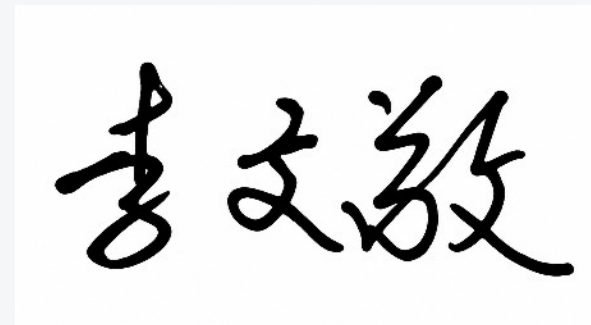
Date	9 September 2026
